# Analysis of motion characteristics and dynamic modeling of traction machine brakes stopping car

**DOI:** 10.1038/s41598-025-99443-5

**Published:** 2025-05-24

**Authors:** Weimin Zhang, Wenxin Zhang, Lizhong Ye, Chengjun Wu

**Affiliations:** 1 School of Mechanical Engineering, Xi’an Jiaotong University, Xi’an, 710049 Shaanxi China; 2 Zhejiang Zynoe Elevator Parts Co., Ltd., Jiaxing, 314423 Zhejiang China; 3 Zhejiang Academy of Special Equipment Science, Hangzhou, 310009 Zhejiang China

**Keywords:** Elevator, Brake, Rope, Slip, Traction, UCMP, Mechanical engineering, Applied mathematics

## Abstract

Braking distance is one of the important indicators for measuring elevator safety. At present, using the traction machine brakes to stop the traction sheave and indirectly stop the car is the mainstream stopping method for elevators with gearless machine. However, in existing theoretical research, the complex elevator braking process is only modeled through simple constant acceleration motion, without considering the influence of the sealing device, let alone the sliding motion of the suspension ropes on the traction sheave after the traction sheave stops rotating, the calculated results are much smaller than the actual situation, which poses a huge challenge to the safe operation of the elevator. Therefore, this article analyzes the slip dynamics characteristics of the suspension ropes on the traction sheave and the asynchronous operation characteristics of traction shave and car during the car braking process under the influence of nonlinear sealing torque and braking torque. A new differential equation for acceleration, velocity, and displacement of the car is established, and the equation is solved through approximate methods. The experimental results show that the theoretical model proposed in this paper can accurately evaluate the dynamic characteristics of the car braking process and has wide engineering application value.

## Introduction

**Fig. 1:**
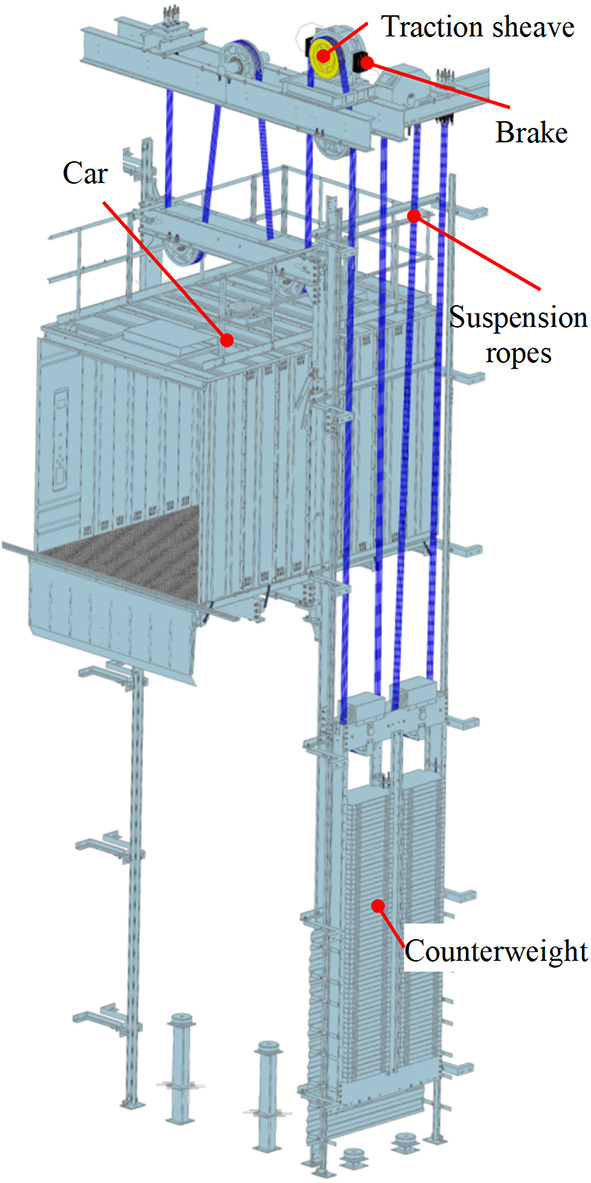
Elevator schematic diagram. Software information for generating Figure: Software Name: ZW3D 2023Xx64 (27.30). Version number: 08/25/2022. URL link: https://www.zwsoft.com/.

With the increasing number of elevators, people are paying more and more attention to their safety. At present, the transmission method of elevators mainly adopts traction type. During normal use of elevators, the friction (traction force) between the groove of the traction sheave of traction machine and the suspension ropes pulls the car and the counterweight vertically up and down on both sides, as shown in Fig. 1. When there are unexpected situations such as emergency braking, ascend car overspeed, and unintended car movement, the car is stopped by relying on the rope griper or traction machine brakes. Therefore, the stopping method can be divided into two types: direct acting type and indirect acting type.

**Fig. 2:**
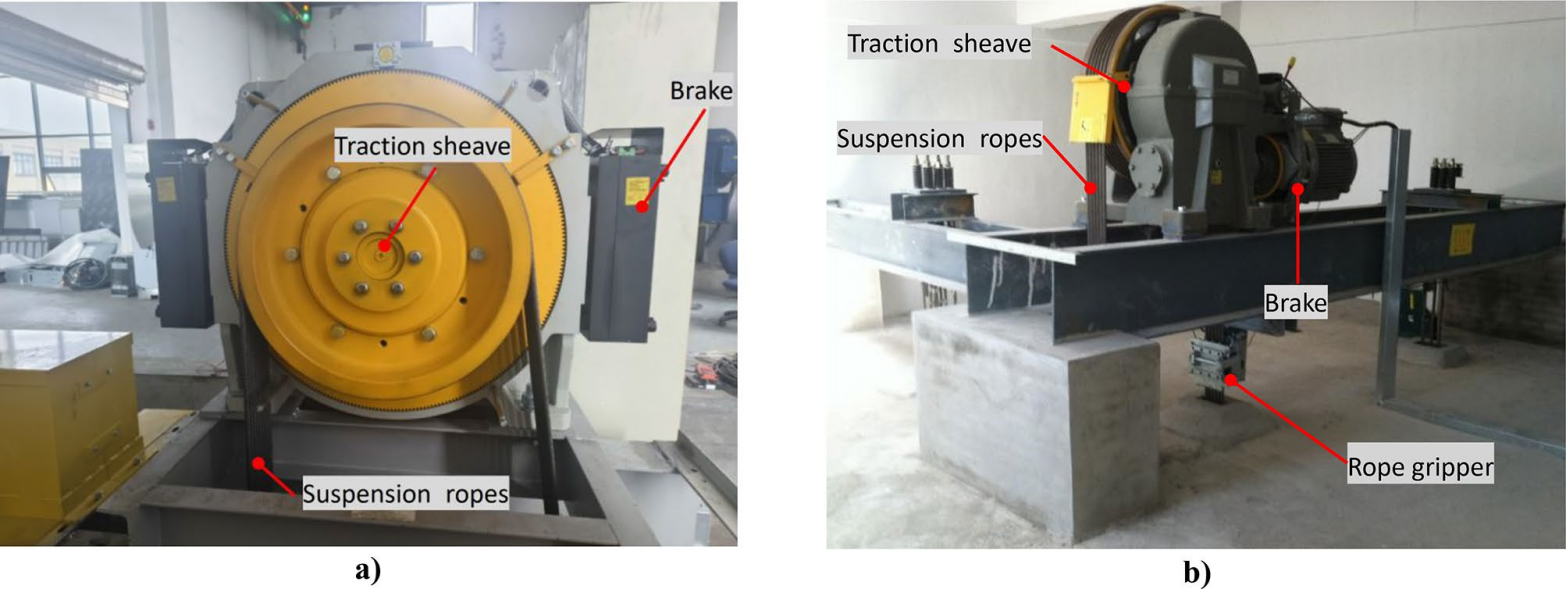
Schematic diagram of action mode (**a**) Indirect action mode (**b**) Direct action mode.

- Indirect action type: as shown in Fig. 2a), the traction sheave is stopped by the brakes on the traction machine, and then the car is stopped by the traction force.
- Direct action type: As shown in Fig. 2b), the car is stopped by clamping the suspension ropes with a rope gripper.

Depending on the type of traction machine, the braking method also varies, mainly as follows:

- Emergency braking condition (EBC): The protective measures taken by elevators during normal operation, such as sudden power outages or safety circuit disconnections, regardless of whether the elevator uses a gear machine or a gearless machine, are indirect acting type.
- Ascend car overspeed (ACO): The elevator runs upwards at an overspeed until it reaches the speed limit of speed governor^1^. For elevators that use gearless engines, the brakes on the traction machine is generally used as an overspeed protection device for the ascend car movement, so an indirect action type is basically adopted. While for elevators using geared machine, some use direct action type and some use indirect action type.
- Unintended car movement (UCM): Protective measures to prevent the car from leaving the landing without receiving any instructions when the car door is not closed^2–4^. For elevators that use gearless main engines, the brakes on the traction machine is generally used as the braking subsystem, so an indirect action type is basically adopted^5^, while for elevators using geared machine, some use direct action type and some use indirect action type.

If the elevator car moves beyond the expected distance after the above three working conditions occur, it will bring serious consequences such as equipment damage, personnel injury, and even death^6–12^. At present, most elevators use gearless main machine, which indirectly stop the car by stopping the traction sheave through the traction machine brakes. The deceleration of the traction sheave cannot be simply used as the deceleration of the car, which will result in calculation results far smaller than the actual stopping distance and serious design defects. Therefore, dynamic analysis during the stopping process is very important.

Some scholars have analyzed the use of the suspension ropes as a means to prevent micro slip caused by severe wear on the traction sheave groove or uneven tension on both sides of the suspension ropes during long-term or abnormal use^13–15^, which is not the dynamic slip situation of the suspension ropes analyzed in this article.

At present, there is very little research and analysis on the sliding dynamics of the suspension ropes that rely on traction force to stop elevator car at a certain speed in the elevator field. Some scholars have studied the dynamic friction transmission and creep characteristics between ropes and friction linings^15–18^, Youngkiu Choi et al. studied the calculation method of direct action equations^19^. Some scholars do not consider the weight of the suspension ropes, the weight of the compensating chain, and the inertia of the pulley in the calculation process of emergency braking conditions^20,21^. The mainstream calculation method for the car stopping distance of the car using indirect action protection devices is to directly use the deceleration of the traction sheave as the deceleration of the car(Abbreviation: OM)^22–28^.

In short, there is very little research on the dynamics of the gross slip of the suspension ropes on the traction sheave when the elevator relies on the traction force to stop the car at a certain speed. They did not take into account that the deceleration of the car is a non-constant value, and the friction coefficient between the suspension ropes and the traction sheave is also a speed dependent variable. They also did not consider the influence of the sealing torque directly related to the speed of traction shave, the nonlinear loading of the braking torque, and the further slippage of the suspension ropes on the traction sheave after the traction sheave stops rotating. The current method ignores the nonlinearity of the car’s deceleration as a constant value when calculating emergency braking condition, ascend car overspeed, and unintended car movement distance, resulting in significant errors and serious safety hazards.

## Explanation of EBC, ACO and UCM

The Chinese elevator code TSG T7007-2022 has added relevant requirements for "other braking devices (functions) of traction driven elevators"^29^, and elevator manufacturers generally choose to add sealing devices. Therefore, for most elevators currently, in the event of emergency braking conditions, ascend car overspeed and unintended car movement, the main protective measures rely on the braking force provided by the brakes and the reverse torque provided by the sealing torque^30–32^.

Note: Sealing torque refers to the mechanical torque generated by the movement of the car and counterweight, which drives the traction machine to rotate into a generator. The electromagnetic torque generated by the electromagnetic induction of the generator interacts with the magnetic force of the permanent magnet, resulting in a braking torque that is opposite to the rotation direction of the traction sheave and related to the speed.

**Fig. 3:**
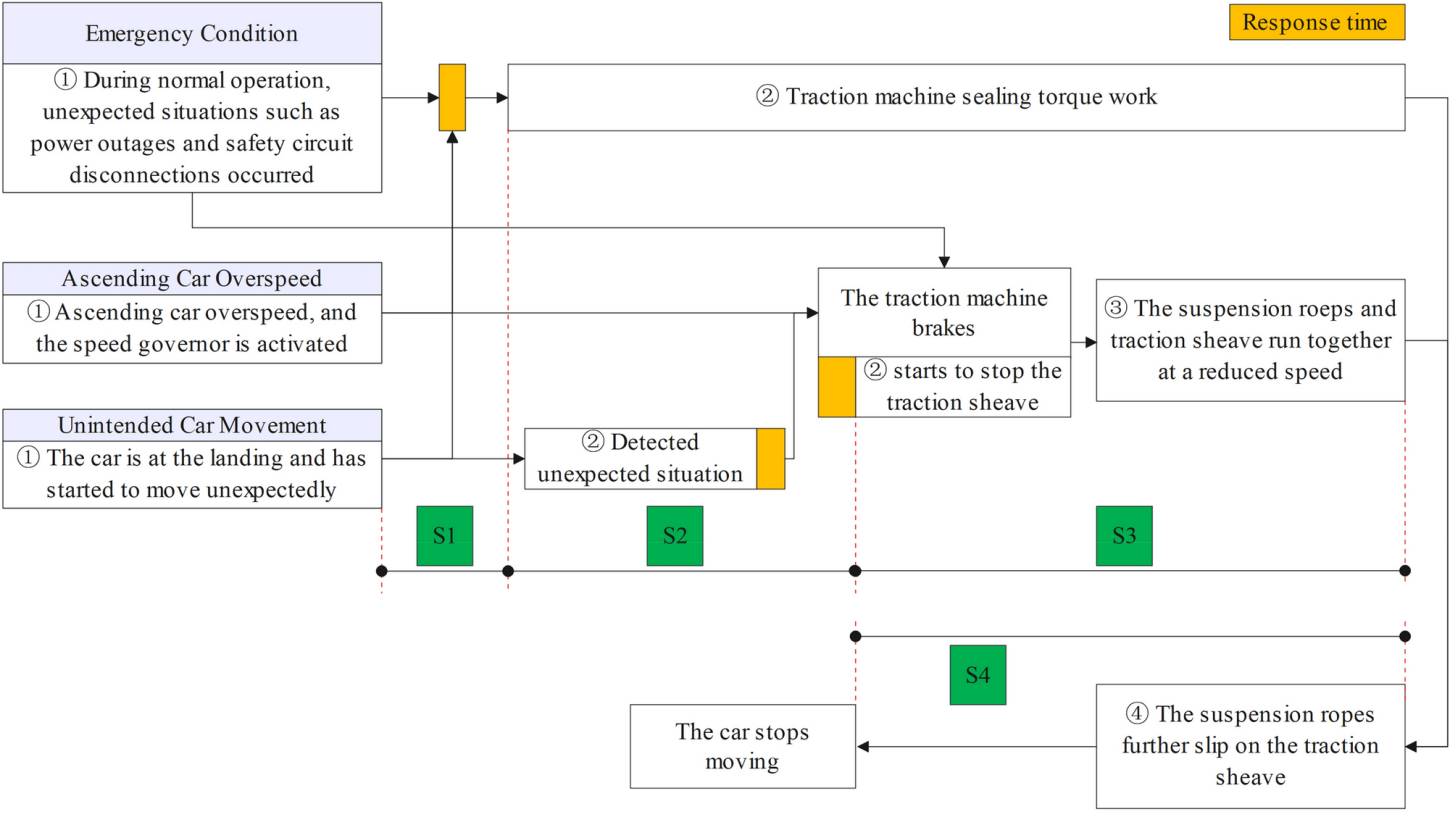
Timing diagram.

The process from the occurrence of emergency braking conditions, ascend car overspeed and unintended car movement, to the activation of protective devices, and finally achieving car stop is basically the same, with some minor differences. The timing diagram is shown in Fig. 3.

Based on Fig. 3, the specific description of the occurrence process of the three working conditions is as follows:

- Emergency braking condition.

The general process of emergency braking conditions is as follows:

① The elevator car is running at rated speed and suddenly encounters unexpected situations such as power outage and safety circuit disconnection:
② After about a few tens of milliseconds (some manufacturers use time judgment, while others use traction sheave rotation angle judgment), the sealing torque begins to take effect.
② After approximately 200 ms of brake response time, the brakes are ready to start stopping the traction sheave.
③ The suspension ropes and traction sheave perform deceleration motion together until the traction sheave stops rotating.
④ The suspension ropes further slides on the traction sheave and finally stops sliding by the traction force, achieving the stopping of the car movement.

Note: Two ② indicate simultaneous occurrence.

(b) Ascend car overspeed.

This condition only occurs when the car is going up, because when the car is going down and overspeed occurs, the final protective device is the safety, which is irrelevant to the analysis in this article. The process is roughly as follows:

The unexpected situation of overspeed occurs when the elevator car runs upwards, until the speed reaches the action speed of the speed governor. The subsequent process is consistent with steps ②–④ during the emergency braking condition.

(c) Unintended car movement.
  ① The elevator car stopped in landing and accidentally left the landing station, followed by the following two steps occurring simultaneously.
  ② After about a few tens of milliseconds, the sealing torque begins to take effect.
  ③ The detection subsystem detects unexpected movement of the car and issues a braking command to the traction machine brakes, and then the brakes prepares to start stopping the traction sheave.

The subsequent process is consistent with steps ③ and ④ during the occurrence of emergency braking conditions.

## Theoretical model

### Theoretical model of motion for traction sheave

#### The elevator car moves upwards

The schematic diagram of the elevator moving up or down shows that the velocity and acceleration on the traction sheave acting on the car need to be divided by the reeving factor *r*^33^. In order to describe and calculate uniformly, this article defines:

- Reduced acceleration of the traction sheave: the acceleration of the traction sheave divided by the reeving factor *r*.
- Reduced velocity of the traction sheave: the linear velocity of the traction sheave divided by the reeving factor *r*.

**Fig. 4:**
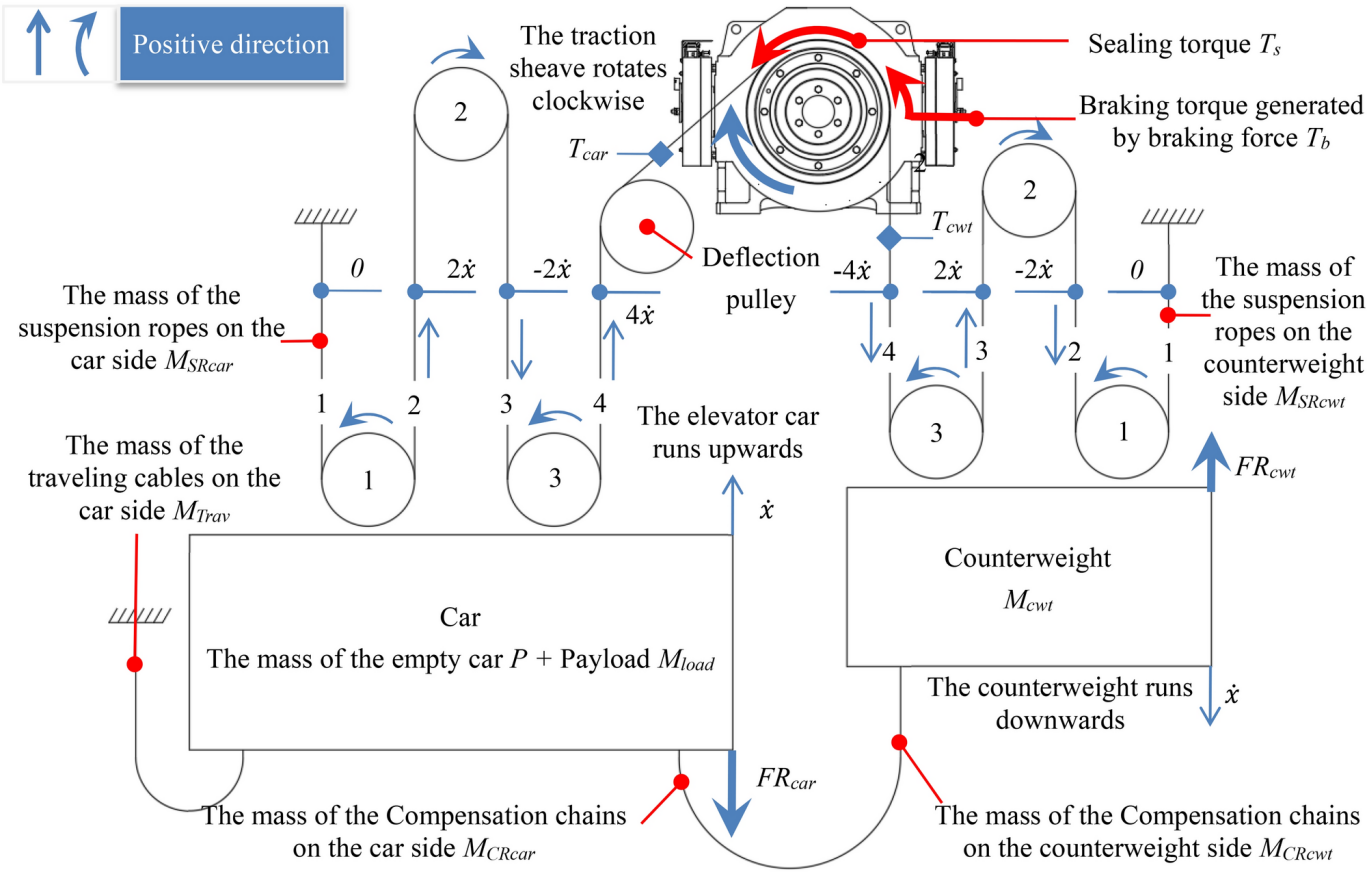
Car motion model diagram.

The simplified model of elevator motion is shown in Fig. 4. From Fig. 4, it can be seen that when the elevator runs upwards, the counterweight runs downwards at the same speed, and the rotational speed of all guide pulleys is related to the speed of the car,. Therefore, this system is a system with a degree of freedom of 1. The displacement of the car *x* is defined as the generalized coordinate, and the kinetic and potential energy of the system are analyzed.

When the tension of the suspension ropes on the car side is less than that on the counterweight side, the sliding distance of the suspension ropes is relatively large under emergency braking, ascend car movement and unintended car movement conditions when the car is running upwards, and vice versa. Therefore, it is only necessary to analyze these two situations. In short, it is:

- When the velocity direction of the car is upward, only the situation where the tension of the suspension ropes on the car side is lower than that on the counterweight side needs to be analyzed.
- When the velocity direction of the car is downward, only the situation where the tension of the suspension ropes on the car side is greater than that on the counterweight side needs to be analyzed.

According to Fig. 4, calculate the kinetic energy and potential energy.

- Kinetic energy calculation.

The kinetic energy of the system includes the kinetic energy of linear moving components, such as the masses of the empty car *P*, the payload *M*_*load*_, the mass of the counterweight *M*_*cwt*_, the mass of the compensation chain on the car side *M*_*CRcar*_, the mass of the compensation chain on the counterweight side *M*_*CRcwt*_, the mass of the traveling cable on the car side *M*_*Trav*_, the kinetic energy of the suspension ropes on both sides of the traction sheave *E*_*SR*_, and the kinetic energy of rotating components, such as the kinetic energy generated by the pulleys. From Fig. 4, it can be concluded that if the elevator reeving factor *r* is greater than 1, there are one or more pulleys on both the car side and the counterweight side. Due to the correlation between kinetic energy and the square of velocity, only their values need to be calculated. The rotational velocity of the i-th pulley on the car side |*ω*_*pcar*-i_| is (*i*/*R*_*pcar-i*_)*dx*/*dt*, and the rotational velocity of the i-th pulley on the counterweight side |*ω*_*pcwt*-i_| is (*i*/*R*_*pcwt-i*_)*dx*/*dt*. Define the velocity of the i-th section of the suspension ropes on the car side as *v*_*SRcar-i*_ and the velocity of the i-th section of the suspension ropes on the counterweight side as *v*_*SRcwt-i*_, as follows:

1 $$\left| {\mathop v\nolimits_{SRcar - i} } \right| = \left| {\mathop v\nolimits_{SRcwt - i} } \right| = \left\{ {\begin{array}{*{20}l} {i\frac{dx}{{dt}}} \hfill & {{\text{if}}\;i\;\bmod \;2 = 0} \hfill \\ {\left( {i - 1} \right)\frac{dx}{{dt}}} \hfill & {{\text{if}}\;i\;\bmod \;2 = 1} \hfill \\ \end{array} } \right.$$

From Fig. 4, it can be concluded that the number of suspension ropes segments on both sides of the traction sheave is equal to the value of the reeving factor *r*. Based on the velocity of each suspension ropes segment in Eq. (1), the kinetic energy of the suspension ropes on both sides of the traction shave *E*_*SR*_ can be obtained, and simplified by induction to obtain the following equation^34^:

2 $$\mathop E\nolimits_{SR} = \frac{1}{2}\sum\limits_{i = 1}^{r} {\left( {\mathop M\nolimits_{SRcar} \mathop v\nolimits_{SRcar - i}^{2} \mathop { + M}\nolimits_{SRcwt} \mathop v\nolimits_{SRcwt - i}^{2} } \right)} = \frac{1}{2}\left( {\mathop M\nolimits_{SRcar} \mathop { + M}\nolimits_{SRcwt} } \right)r\frac{{\left( {\mathop r\nolimits^{2} + 2} \right)}}{3}\mathop {\left( {\frac{dx}{{dt}}} \right)}\nolimits^{2}$$

The equation for the kinetic energy of the system *E* is as follows:

3 $$\begin{gathered} E = \frac{1}{2}\left( {P + \mathop M\nolimits_{load} + \mathop M\nolimits_{cwt} + \mathop M\nolimits_{CRcar} + \mathop M\nolimits_{CRcwt} + \mathop M\nolimits_{Trav} } \right)\left( {\frac{dx}{{dt}}} \right)^{2} + \frac{{\mathop J\nolimits_{DP} }}{2}\left( {\frac{r}{{\mathop R\nolimits_{DP} }}\frac{dx}{{dt}}} \right)^{2} \hfill \\ \;\;\;\;\;\; + \frac{r}{6}\left( {\mathop r\nolimits^{2} + 2} \right)\left( {\mathop M\nolimits_{SRcar} \mathop { + M}\nolimits_{SRcwt} } \right)\mathop {\left( {\frac{dx}{{dt}}} \right)}\nolimits^{2} + \frac{{sign\left( {r - 1} \right)}}{2}\sum\limits_{i = 1}^{r - 1} {\left( {\frac{{\mathop J\nolimits_{Pcar - i} \mathop n\nolimits_{Pcar - i} }}{{\mathop R\nolimits_{Pcar - i}^{2} }} + \frac{{\mathop J\nolimits_{Pcwt - i} \mathop n\nolimits_{Pcwt - i} }}{{\mathop R\nolimits_{Pcwt - i}^{2} }}} \right)\left( {i\frac{dx}{{dt}}} \right)^{2} } \hfill \\ \end{gathered}$$

(b) Potential energy calculation.

The total potential energy *U* of the system is as follows:

The total potential energy of the system *U* includes the potential energy generated by the vertical position changes of the masses of the empty car *P*, the payload *M*_*load*_, the mass of the counterweight *M*_*cwt*_, the mass of the compensation chain on the car side *M*_*CRcar*_, the mass of the compensation chain on the counterweight side *M*_*CRcwt*_ and the mass of the traveling cable on the car side *M*_*Trav*_, as well as the dissipation force generated by the frictional force in the well on the car side *F*_*rcar*_, the frictional force in the well on the counterweight side *F*_*rcwt*_, the braking torque *T*_*b*_ and sealing torque *T*_*s*_ of the traction machine brake acting on the traction sheave. Since the rotation distance of the traction sheave is *r* times the movement distance of the car, when calculating the potential energy of the braking torque *T*_*b*_ and sealing torque *T*_*s*_*,* it needs to be multiplied by *r*, the specific formula is as follows:

4 $$U = \mathop {xg}\nolimits_{n} \left[ \begin{gathered} \mathop M\nolimits_{cwt} + \mathop M\nolimits_{CRcwt} + \mathop {rM}\nolimits_{SRcwt} - P - \mathop M\nolimits_{load} - \mathop M\nolimits_{CRcar} \hfill \\ - \mathop {rM}\nolimits_{SRcar} - \mathop M\nolimits_{Trav} - r\frac{{\mathop T\nolimits_{b} + \mathop T\nolimits_{s} }}{{\mathop R\nolimits_{t} }} + \frac{{\mathop {FR}\nolimits_{car} - \mathop {FR}\nolimits_{cwt} }}{{\mathop g\nolimits_{n} }} \hfill \\ \end{gathered} \right]$$

(c) Dynamic equation.

The Lagrange equation is as follows:

5 $$\frac{d}{dt}\left( {\frac{{\partial \left( {E - U} \right)}}{{\partial \left( {\frac{dx}{{dt}}} \right)}}} \right) - \frac{{\partial \left( {E - U} \right)}}{\partial x} = 0$$

For the convenience of calculating the tension of the suspension ropes on both sides of the traction sheave, Eqs. (3) and (4) are simultaneously divided by the reeving factor *r* and substituted into Eq. (5) to obtain:

6 $$\left( {\mathop A\nolimits_{car} + \mathop A\nolimits_{cwt} } \right)\frac{{d^{2} x}}{{dt^{2} }} + \mathop B\nolimits_{car} + \frac{{\mathop T\nolimits_{b} + \mathop T\nolimits_{s} }}{{\mathop R\nolimits_{t} }} - \mathop B\nolimits_{cwt} = 0$$

where

$$\left\{ \begin{gathered} \mathop A\nolimits_{car} = \frac{{\left( {P + \mathop M\nolimits_{load} + \mathop M\nolimits_{CRcar} + \mathop M\nolimits_{Trav} } \right)}}{r} + \mathop M\nolimits_{SRcar} \left( {\frac{{\mathop r\nolimits^{2} + 2}}{3}} \right) + \left[ {\frac{{\mathop {rJ}\nolimits_{DP} }}{{\mathop R\nolimits_{DP}^{2} }}} \right]^{{\text{I}}} + \frac{{sign\left( {r - 1} \right)}}{r}\sum\limits_{i = 1}^{r - 1} {\frac{{\mathop J\nolimits_{Pcar - i} \mathop n\nolimits_{Pcar - i} }}{{\mathop R\nolimits_{Pcar - i}^{2} }}i^{2} } \hfill \\ \mathop B\nolimits_{car} = \frac{1}{r}\left[ {\left( {P + \mathop M\nolimits_{load} + \mathop M\nolimits_{CRcar} + \mathop M\nolimits_{Trav} + \mathop {rM}\nolimits_{SRcar} } \right)\mathop g\nolimits_{n} - \mathop {FR}\nolimits_{car} } \right] \hfill \\ \mathop A\nolimits_{cwt} = \frac{{\left( {\mathop M\nolimits_{cwt} + \mathop M\nolimits_{CRcwt} } \right)}}{r} + \mathop M\nolimits_{SRcwt} \left( {\frac{{\mathop r\nolimits^{2} + 2}}{3}} \right) + \left[ {\frac{{\mathop {rJ}\nolimits_{DP} }}{{\mathop R\nolimits_{DP}^{2} }}} \right]^{{\text{I}}} + \frac{{sign\left( {r - 1} \right)}}{r}\sum\limits_{i = 1}^{r - 1} {\frac{{\mathop J\nolimits_{Pcwt - i} \mathop n\nolimits_{Pcwt - i} }}{{\mathop R\nolimits_{Pcwt - i}^{2} }}i^{2} } \hfill \\ \mathop B\nolimits_{cwt} = \frac{1}{r}\left[ {\left( {\mathop M\nolimits_{cwt} + \mathop M\nolimits_{CRcwt} + \mathop {rM}\nolimits_{SRcwt} } \right)\mathop g\nolimits_{n} - \mathop {FR}\nolimits_{cwt} } \right] \hfill \\ \end{gathered} \right.$$

Conditions:

I is for any deflection pulley on car side.

II is for any deflection pulley on counterweight side.

By organizing Eq. (6), the calculation equation for the reduced acceleration of the traction sheave *am-u* when the elevator ascends is as follows:

7 $$\mathop a\nolimits_{m - u} = \frac{{d^{2} x}}{{dt^{2} }} = \frac{{\mathop B\nolimits_{cwt} - \mathop B\nolimits_{car} - \left( {\frac{{\mathop T\nolimits_{b} + \mathop T\nolimits_{s} }}{{\mathop R\nolimits_{t} }}} \right)}}{{\mathop A\nolimits_{car} + \mathop A\nolimits_{cwt} }}$$

#### The elevator car moves downwards

When the car moves downwards, the calculation method for the reduced acceleration of the traction sheave is similar to that for the upward movement of the car. Therefore, the calculation equation for the reduced acceleration of the traction sheave *a*_*m-d*_ is derived as follows:

8 $$\mathop a\nolimits_{m - d} = \frac{{d^{2} x}}{{dt^{2} }} = \frac{{\mathop {\mathop B\nolimits_{cwt} - B}\nolimits_{car} + \frac{{\mathop T\nolimits_{b} + \mathop T\nolimits_{s} }}{{\mathop R\nolimits_{t} }}}}{{\mathop A\nolimits_{car} + \mathop A\nolimits_{cwt} }}$$

### Unified equation

For the convenience of description and calculation, Eqs. (7) and (8) can be expressed as one equation, with the direction symbol *k*_*s*_. When the velocity of the car is a positive, *k*_*s*_ = 1, conversely, *k*_*s*_ = − 1, as follows:

9 $$\mathop k\nolimits_{s} = sign\left( V \right)$$

Equations (7) and (8) only have different symbols, so they can be combined into one equation as follows.

10 $$\mathop a\nolimits_{m} = \frac{{d^{2} x}}{{dt^{2} }} = \frac{{\mathop B\nolimits_{cwt} - \mathop B\nolimits_{car} - \frac{{\mathop k\nolimits_{s} }}{{\mathop R\nolimits_{t} }}\left( {\mathop T\nolimits_{b} + \mathop T\nolimits_{s} } \right)}}{{\mathop A\nolimits_{car} + \mathop A\nolimits_{cwt} }}$$

### Sliding conditions of the suspension ropes on traction sheave

**Fig. 5:**
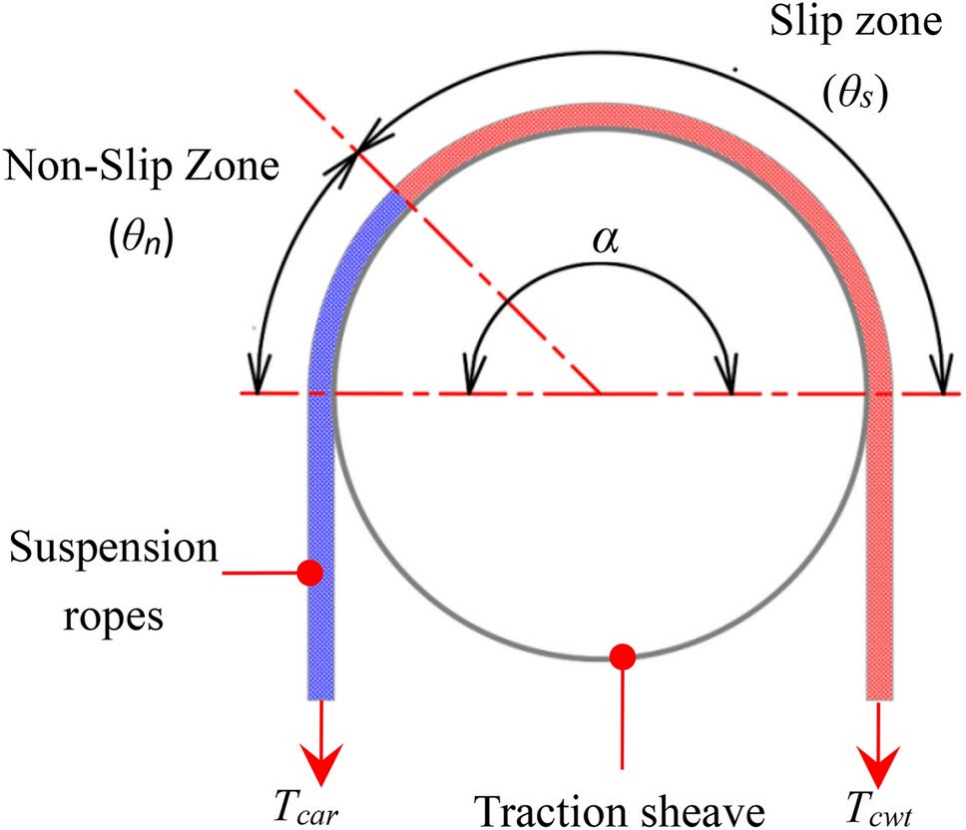
Diagram of the state of the suspension ropes before gross slip on the traction sheave.

Figure 5 shows a schematic diagram of hanging suspension ropes on traction sheave, The angle of wrap of the suspension ropes on the traction sheave is *α,* according to the previous definition:

- When the elevator car is running upwards, the tension of the suspension ropes on the left side *T*_*car*_ is less than the tension of the suspension ropes on the right side *T*_*cwt*_.
- When the car is running downwards, the tension of the suspension ropes on the left side *T*_*car*_ is greater than the tension of the suspension ropes on the right side *T*_*cwt*_.

According to the Euler–Eytelwein equation, the contact area between the suspension ropes and the traction sheave can be divided into two zones: slip zone and non-slip zone^16–19,35,36^. When the non-slip angle *θ*_*n*_ is not less than 0°, the suspension ropes is in the creep stage, otherwise it enters the gross slip stage^37,38^. The Non slip angle *θ*_*n*_ is related to the equivalent friction coefficient between suspension ropes and traction sheave *f,* the angle of wrap of the ropes on the traction sheave *α*, and the tension of the suspension ropes on both sides of the traction sheave, the specific calculation formula is as follows:

11 $$\mathop \theta \nolimits_{n} = \alpha - \mathop \theta \nolimits_{s} = \alpha - \frac{1}{f}\lg \left( {\frac{{\mathop T\nolimits_{cwt} }}{{\mathop T\nolimits_{car} }}} \right)^{{k_{s} }} = \alpha - \frac{1}{f}\lg \left( {\frac{{\mathop {\mathop B\nolimits_{cwt} - A}\nolimits_{cwt} \mathop a\nolimits_{e} }}{{\mathop B\nolimits_{car} + \mathop A\nolimits_{car} \mathop a\nolimits_{e} }}} \right)^{{\mathop k\nolimits_{s} }}$$

When the weight on the car side is greater than the counterweight side, regardless of whether the elevator is running up or down, the suspension ropes will creep towards the car side; On the contrary, when the weight of the counterweight is greater than that of the car, the suspension ropes will creep towards the counterweight side. Therefore, the elastic slip of the suspension ropes always crawls towards the side with heavier load, and the creep velocity is related to the weight difference *ΔT* on both sides of the traction sheave, the elastic modulus *E*_*r*_ and area *A*_*r*_ of the suspension ropes, and the velocity of the suspension ropes *rv*_*e*_^39^, the specific calculation formula is as follows:

12 $$\mathop v\nolimits_{c} = \frac{1}{r}\frac{\Delta T}{{\mathop E\nolimits_{r} \mathop A\nolimits_{r} }}\mathop {rv}\nolimits_{e} = \frac{{\mathop k\nolimits_{s} \left( {\mathop T\nolimits_{cwt} - \mathop T\nolimits_{car} } \right)}}{{\mathop E\nolimits_{r} \mathop A\nolimits_{r} }}\mathop v\nolimits_{e} = \frac{{\mathop {4k}\nolimits_{s} \left( {\mathop {\mathop B\nolimits_{cwt} - A}\nolimits_{cwt} \mathop a\nolimits_{e} - \mathop B\nolimits_{car} - \mathop A\nolimits_{car} \mathop a\nolimits_{e} } \right)}}{{\mathop {\pi E}\nolimits_{r} d_{r}^{2} }}\mathop v\nolimits_{e}$$

From Eq. (12), it can be seen that the creep velocity is almost negligible compared to the car velocity *ve*, so this article only calculates the gross slip distance of the suspension ropes.

From Eq. (11), it can be concluded that when *θ*_*n*_ = 0, the traction force of the elevator can provide the maximum acceleration of the car. According to the definition of the friction coefficient *f* between the suspension ropes and the traction sheave in clause 5.11.2.3 of elevator code EN 81-50:2014^40^ and GB/T 7588.2-2020^41^, and with reference to the research on the friction coefficient between the suspension ropes and the traction sheave by other scholars^42,43^, the relationship between the acceleration at provided by the elevator traction force and the relative velocity *v*_*rs*_ between the suspension ropes and the traction sheave is obtained as follows:

13 $$\left( {\frac{{\mathop T\nolimits_{cwt} }}{{\mathop T\nolimits_{car} }}} \right)^{{\mathop k\nolimits_{s} }} = \left( {\frac{{\mathop {\mathop B\nolimits_{cwt} - A}\nolimits_{cwt} \mathop a\nolimits_{e} }}{{\mathop B\nolimits_{car} + \mathop A\nolimits_{car} \mathop a\nolimits_{e} }}} \right)^{{\mathop k\nolimits_{s} }} = e^{{\frac{\lambda }{{10 + \mathop k\nolimits_{s} \mathop v\nolimits_{rs} }}}}$$

where

$$\lambda = 4\alpha \frac{{\cos \left( {\frac{\gamma }{2}} \right) - \sin \left( {\frac{\beta }{2}} \right)}}{\pi - \beta - \gamma - \sin \left( \beta \right) + - \sin \left( \gamma \right)}$$

By organizing Eq. (13), the calculation equation for the maximum car acceleration *a*_*t*_ provided by the traction force is as follows:

14 $$\mathop a\nolimits_{t} = \mathop k\nolimits_{s} \left( {\frac{{\left( {1 + \mathop k\nolimits_{s} } \right)\mathop B\nolimits_{car} }}{{2\mathop A\nolimits_{car} }} + \frac{{\mathop {\left( {1 - \mathop k\nolimits_{s} } \right)B}\nolimits_{cwt} }}{{\mathop {2A}\nolimits_{cwt} }}} \right)\left( {\frac{{\left( {\frac{{\mathop A\nolimits_{cwt} }}{{\mathop A\nolimits_{car} }}} \right)^{{\mathop k\nolimits_{s} }} + \left( {\frac{{\mathop B\nolimits_{cwt} }}{{\mathop B\nolimits_{car} }}} \right)^{{\mathop k\nolimits_{s} }} }}{{e^{{\frac{\lambda }{{10 + \left| {r\mathop v\nolimits_{rs} } \right|}}}} + \left( {\frac{{\mathop A\nolimits_{cwt} }}{{\mathop A\nolimits_{car} }}} \right)^{{\mathop k\nolimits_{s} }} }} - 1} \right)$$

The difference between the reduced acceleration of the traction sheave *a*_*m*_ calculated by Eq. (10) and the maximum car acceleration *at* provided by the traction force calculated by Eq. (14) yields the following car acceleration:

- Before the traction sheave stops rotating:
  1. If |*a*_*t*_| >|*a*_*m*_|, the speed of the car decreases faster than the reduced speed of the traction sheave, and the suspension ropes does not slip on the traction sheave. However, since the traction sheave has not stopped rotating, it will drive the car to move. Therefore, the acceleration of the car *a*_*e*_ is equal to the reduced acceleration of the traction sheave *a*_*m*_.
  2. If |*a*_*t*_| ≤|*a*_*m*_|, the decrease in the speed of the car is slower than the decrease in the reduced speed of the traction sheave, and the suspension ropes slip on the traction sheave. Therefore, the acceleration of the car *a*_*e*_ is equal to the car acceleration *a*_*t*_ that provided by the traction force.
- After the traction sheave stops rotating

If the car speed is greater than 0 after the previous process is completed, then the car acceleration *a*_*e*_ is the acceleration *a*_*t*_ provided by the traction force.

It should be noted that when the signs of *a*_*m*_ and *a*_*t*_ are opposite, that is, when the car is accelerating at system acceleration, the difference in weight between the car and the counterweight drives the traction sheave to accelerate, so it cannot be judged according to the above conditions.

### Dynamical model

According to Sect.  2, the entire process of the three operating conditions, namely emergency braking conditions, ascend car overspeed and unintended car movement, is divided into four stages:

- Free acceleration stage (S1 stage).
- Sealing torque intervention stage (S2 stage).
- Brakes stopping traction sheave stage (S3 stage).
- Suspension ropes further sliding stage (S4 stage).

#### Free acceleration stage (S1 stage)

At this stage, the principles of emergency braking condition, ascend car overspeed and unintended car movement are the same, except for the initial velocity, the initial speeds are:

- Emergency braking condition: rated speed of elevator *V*.
- Ascend car overspeed: speed limiter action speed *V*_*sg*_.
- Unintended car movement 0 m/s.

The specific formula is as follows:

15 $$\mathop V\nolimits_{e0} = \mathop V\nolimits_{m0} = \left\{ {\begin{array}{*{20}l} V \hfill & {{\text{EBC}}} \hfill \\ {\mathop V\nolimits_{sg} } \hfill & {{\text{ACO}}} \hfill \\ 0 \hfill & {{\text{UCM}}} \hfill \\ \end{array} } \right.$$

Due to the fact that the sealing torque has not yet been applied and the brakes has not started to stop the traction sheave, both the braking torque and star sealing torque are 0 at this stage, and the car and traction sheave are accelerating freely synchronously. Substitute the initial velocity in Eq. (15) into Eq. (10) to obtain the acceleration of the car *a*_*e*_ and the reduced acceleration of the traction sheave *a*_*m*_. Based on the acceleration, the equations for the car velocity *v*_*e*_, reduced velocity of the traction sheave *v*_*m*_, and car displacement *x*_*e*_ are as follows:

16 $$\left\{ \begin{gathered} \mathop a\nolimits_{e} = \mathop a\nolimits_{m} = \frac{{\mathop B\nolimits_{cwt} - \mathop B\nolimits_{car} }}{{\mathop A\nolimits_{car} + \mathop A\nolimits_{cwt} }} \hfill \\ \mathop v\nolimits_{e} = \mathop V\nolimits_{e0} + \mathop a\nolimits_{e} t \hfill \\ \mathop x\nolimits_{e} = \mathop V\nolimits_{e0} t + \frac{1}{2}\mathop a\nolimits_{e} t^{2} \hfill \\ \end{gathered} \right.$$

The running time of this stage *t*_1_ is the sealing torque response time *t*_*st*_. According to the above equation, the acceleration of the car *a*_*e*1_, the reduced acceleration of the traction sheave *a*_*m*1_, the velocity of the car *v*_*e*1_, the reduced velocity of the traction sheave *v*_*m*1_, and the car displacement *x*_*e*1_ can be obtained after the end of this stage.

#### Sealing torque intervention stage (S2 stage)

**Fig. 6:**
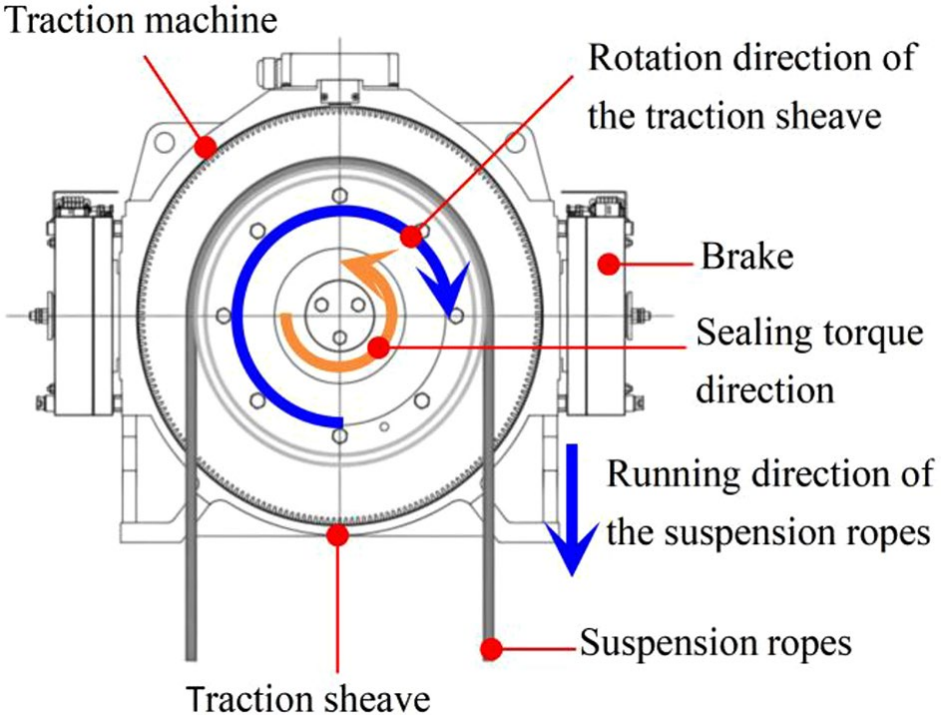
Operation status diagram.

At this stage, the sealing torque begins to intervene, and its direction of action is always opposite to the rotation direction of the traction sheave. Figure 6 shows the operating state of the traction sheave when it runs clockwise. At this stage, the brakes have not yet started to stop the traction sheave, so only the sealing torque plays a role in slowing down the traction sheave.

The sealing torque *T*_*s*_ is related to the converted speed of the traction sheave, as well as the stator resistance *R*_*s*_, the number of pole pairs *P*_*m*_, rotor magnetic flux *φ*_*f*_, cross axis inductance *L*_*d*_, and direct axis inductance *L*_*q*_ of the traction machine^44^, as follows:

17 $$\mathop T\nolimits_{s} = \frac{3}{2}\mathop P\nolimits_{m} \frac{{\frac{{\left| {\mathop {rv}\nolimits_{m}\mathop P\nolimits_{m} } \right|}}{{\mathop R\nolimits_{t} }}\mathop \phi \nolimits_{f}^{2} \mathop R\nolimits_{s}^{3} + \left( {\frac{{\left| {\mathop {rv}\nolimits_{m}\mathop P\nolimits_{m} } \right|}}{{\mathop R\nolimits_{t} }}} \right)^{3} \mathop \phi \nolimits_{f}^{2} \mathop R\nolimits_{s} \mathop L\nolimits_{q}^{2} }}{{\left[ {\mathop R\nolimits_{s}^{2} + \left( {\frac{{\mathop {rv}\nolimits_{m}\mathop P\nolimits_{m} }}{{\mathop R\nolimits_{t} }}} \right)^{2} \mathop L\nolimits_{d} \mathop L\nolimits_{q} } \right]^{2} }}$$

**Fig. 7:**
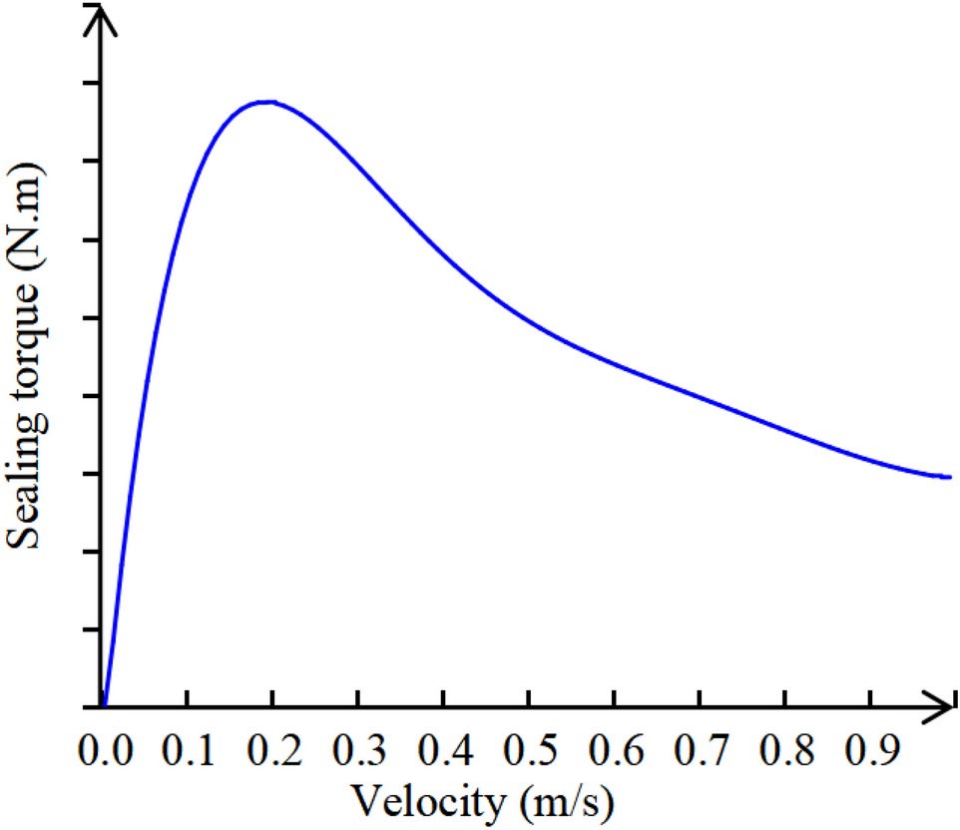
Relationship between sealing torque and speed.

When the reduced velocity of the traction sheave is relatively high or low, the sealing torque is small. At a certain speed in the middle, the sealing torque reaches its maximum value, as shown in Fig. 7.

Substituting the braking torque *T*_*b*_ = 0 and Eq. (17) into Eq. (10), the differential equations for the reduced acceleration *a*_*m*_ and reduced velocity *v*_*m*_ of the traction sheave are obtained as follows:

18 $$\mathop a\nolimits_{m} = \frac{{\mathop {dv}\nolimits_{m} }}{dt} = \frac{{\mathop B\nolimits_{cwt} - \mathop B\nolimits_{car} - \frac{{\mathop {3k}\nolimits_{s} \mathop P\nolimits_{m} }}{{2\mathop R\nolimits_{t} }}\frac{{\left[ {\frac{{\left| {\mathop {rv}\nolimits_{m} \mathop P\nolimits_{m}} \right|}}{{\mathop R\nolimits_{t} }}\mathop \phi \nolimits_{f}^{2} \mathop R\nolimits_{s}^{3} + \left( {\frac{{\left| {\mathop {rv}\nolimits_{m}\mathop P\nolimits_{m} } \right|}}{{\mathop R\nolimits_{t} }}} \right)^{3} \mathop \phi \nolimits_{f}^{2} \mathop R\nolimits_{s} \mathop L\nolimits_{q}^{2} } \right]}}{{\left[ {\mathop R\nolimits_{s}^{2} + \left( {\frac{{\mathop {rv}\nolimits_{m}\mathop P\nolimits_{m} }}{{\mathop R\nolimits_{t} }}} \right)^{2} \mathop L\nolimits_{d} \mathop L\nolimits_{q} } \right]^{2} }}}}{{\mathop A\nolimits_{car} + \mathop A\nolimits_{cwt} }}$$

According to Eqs. (14) and (18), the differential equation for the acceleration of the car *a*_*e*_ is obtained as follows:

19 $$\left\{ {\begin{array}{*{20}l} {\mathop a\nolimits_{e} = \frac{{d^{2} x}}{{dt^{2} }} = \mathop a\nolimits_{t} = \mathop k\nolimits_{s} \left( {\frac{{\left( {1 + \mathop k\nolimits_{s} } \right)\mathop B\nolimits_{car} }}{{2\mathop A\nolimits_{car} }} + \frac{{\mathop {\left( {1 - \mathop k\nolimits_{s} } \right)B}\nolimits_{cwt} }}{{\mathop {2A}\nolimits_{cwt} }}} \right)\left( {\frac{{\left( {\frac{{\mathop A\nolimits_{cwt} }}{{\mathop A\nolimits_{car} }}} \right)^{{\mathop k\nolimits_{s} }} + \left( {\frac{{\mathop B\nolimits_{cwt} }}{{\mathop B\nolimits_{car} }}} \right)^{{\mathop k\nolimits_{s} }} }}{{e^{{\frac{\lambda }{{10 + r\left| {\frac{dx}{{dt}} - \mathop v\nolimits_{m} } \right|}}}} + \left( {\frac{{\mathop A\nolimits_{cwt} }}{{\mathop A\nolimits_{car} }}} \right)^{{\mathop k\nolimits_{s} }} }} - 1} \right)} \hfill & {{\text{if}}\;\left| {\mathop a\nolimits_{t} } \right| \le \left| {\mathop a\nolimits_{m} } \right|} \hfill \\ {\mathop a\nolimits_{e} = \mathop a\nolimits_{m} } \hfill & {{\text{if}}\;\left| {\mathop a\nolimits_{t} } \right| > \left| {\mathop a\nolimits_{m} } \right|} \hfill \\ \end{array} } \right.$$

Due to the fact that Eqs. (18) and (19) cannot directly calculate the original function and have coupling between them, the Explicit Trapezoid Method is used to find an approximate solution. The following equation is an approximate solution method for second-order differential equations, for first-order differential equations, only the second-order term needs to be ignored. Assuming the calculation step size is Δ*t*, the specific calculation equation is as follows:

20 $$\left\{ \begin{gathered} \mathop v\nolimits_{i + 1} = \mathop v\nolimits_{i} + \frac{\Delta t}{2}\left[ {\dot{v}\left( {\mathop t\nolimits_{i} ,\mathop v\nolimits_{i} } \right) + \dot{v}\left( {\mathop t\nolimits_{i} + \Delta t,\mathop v\nolimits_{i} + \Delta t\dot{v}\left( {\mathop t\nolimits_{i} ,\mathop v\nolimits_{i} } \right)} \right)} \right] \hfill \\ \mathop a\nolimits_{i + 1} = \dot{v}\left( {\mathop t\nolimits_{i + 1} ,\mathop v\nolimits_{i + 1} } \right) \hfill \\ \mathop x\nolimits_{i + 1} = \mathop x\nolimits_{i} + \frac{\Delta t}{2}\left[ {\mathop v\nolimits_{i} + \mathop v\nolimits_{i + 1} } \right] \hfill \\ \end{gathered} \right.$$

For Eq. (18), $\dot{v}$ is the equation corresponding to *am*, and the equation has only one independent variable, the reduced velocity of the traction sheave *vm*. Similarly, for Eq. (19), $\dot{v}$ is the equation corresponding to the maximum car acceleration *at* that can be provided by the traction force. Since the equation is related to *ve* and *am*, it has two independent variables *ve* and *vm*. Substitute Eqs. (18) and (19) into Eq. (20) at the same time, and we obtain:

21 $$\left\{ \begin{gathered} \mathop v\nolimits_{{m_{{\begin{array}{*{20}c} {} \\ \end{array} }} i + 1}} = \mathop v\nolimits_{{m_{{\begin{array}{*{20}c} {} \\ \end{array} }} i}} + \frac{\Delta t}{2}\left[ {\mathop a\nolimits_{m} \left( {\mathop v\nolimits_{{m_{{\begin{array}{*{20}c} {} \\ \end{array} }} i}} } \right) + \mathop a\nolimits_{m} \left( {\mathop v\nolimits_{i} + \Delta t\mathop a\nolimits_{m} \left( {\mathop v\nolimits_{{m_{{\begin{array}{*{20}c} {} \\ \end{array} }} i}} } \right)} \right)} \right] \hfill \\ \mathop a\nolimits_{{m_{{\begin{array}{*{20}c} {} \\ \end{array} }} i + 1}} = \mathop a\nolimits_{m} \left( {\mathop v\nolimits_{{m_{{\begin{array}{*{20}c} {} \\ \end{array} }} i + 1}} } \right) \hfill \\ \mathop v\nolimits_{{t_{{\begin{array}{*{20}c} {} \\ \end{array} }} i + 1}} = \mathop v\nolimits_{{e_{{\begin{array}{*{20}c} {} \\ \end{array} }} i}} + \frac{\Delta t}{2}\left[ {\mathop a\nolimits_{t} \left( {\mathop v\nolimits_{{m_{{\begin{array}{*{20}c} {} \\ \end{array} }} i}} ,\mathop v\nolimits_{{e_{{\begin{array}{*{20}c} {} \\ \end{array} }} i}} } \right) + \mathop a\nolimits_{t} \left( {\mathop v\nolimits_{{m_{{\begin{array}{*{20}c} {} \\ \end{array} }} i + 1}} \mathop {,v}\nolimits_{{t_{{\begin{array}{*{20}c} {} \\ \end{array} }} i}} + \Delta t\mathop a\nolimits_{t} \left( {\mathop v\nolimits_{{m_{{\begin{array}{*{20}c} {} \\ \end{array} }} i}} ,\mathop v\nolimits_{{t_{{\begin{array}{*{20}c} {} \\ \end{array} }} i}} } \right)} \right)} \right] \hfill \\ \mathop a\nolimits_{{t_{{\begin{array}{*{20}c} {} \\ \end{array} }} i + 1}} = \mathop a\nolimits_{t} \left( {\mathop v\nolimits_{{m_{{\begin{array}{*{20}c} {} \\ \end{array} }} i + 1}} ,\mathop v\nolimits_{{t_{{\begin{array}{*{20}c} {} \\ \end{array} }} i + 1}} } \right) \hfill \\ \mathop v\nolimits_{{e_{{\begin{array}{*{20}c} {} \\ \end{array} }} i + 1}} = \left\{ {\begin{array}{*{20}c} {\mathop v\nolimits_{{t_{{\begin{array}{*{20}c} {} \\ \end{array} }} i + 1}} } & {{\text{if}}\;\left| {\mathop a\nolimits_{{t_{{\begin{array}{*{20}c} {} \\ \end{array} }} i}} } \right| \le \left| {\mathop a\nolimits_{{m_{{\begin{array}{*{20}c} {} \\ \end{array} }} i}} } \right|} \\ {\mathop v\nolimits_{{m_{{\begin{array}{*{20}c} {} \\ \end{array} }} i + 1}} } & {{\text{if}}\;\left| {\mathop a\nolimits_{{t_{{\begin{array}{*{20}c} {} \\ \end{array} }} i}} } \right| > \left| {\mathop a\nolimits_{{m_{{\begin{array}{*{20}c} {} \\ \end{array} }} i}} } \right|} \\ \end{array} } \right. \hfill \\ \mathop a\nolimits_{{e_{{\begin{array}{*{20}c} {} \\ \end{array} }} i + 1}} = \left\{ {\begin{array}{*{20}c} {\mathop a\nolimits_{{t_{{\begin{array}{*{20}c} {} \\ \end{array} }} i + 1}} } & {{\text{if}}\;\left| {\mathop a\nolimits_{{t_{{\begin{array}{*{20}c} {} \\ \end{array} }} i + 1}} } \right| \le \left| {\mathop a\nolimits_{{m_{{\begin{array}{*{20}c} {} \\ \end{array} }} i + 1}} } \right|} \\ {\mathop a\nolimits_{{m_{{\begin{array}{*{20}c} {} \\ \end{array} }} i + 1}} } & {{\text{if}}\;\left| {\mathop a\nolimits_{{t_{{\begin{array}{*{20}c} {} \\ \end{array} }} i + 1}} } \right| > \left| {\mathop a\nolimits_{{m_{{\begin{array}{*{20}c} {} \\ \end{array} }} i + 1}} } \right|} \\ \end{array} } \right. \hfill \\ \mathop x\nolimits_{{e_{{\begin{array}{*{20}c} {} \\ \end{array} }} i + 1}} = \mathop x\nolimits_{{e_{{\begin{array}{*{20}c} {} \\ \end{array} }} i}} + \frac{\Delta t}{2}\left( {\mathop v\nolimits_{{e_{{\begin{array}{*{20}c} {} \\ \end{array} }} i}} + \mathop v\nolimits_{{e_{{\begin{array}{*{20}c} {} \\ \end{array} }} i + 1}} } \right) \hfill \\ \hfill \\ \end{gathered} \right.$$

The initial parameters in the above equation are the calculation results of Eq. (16) at the end of stage S1, and the acceleration, velocity, and displacement of the car at different times during this stage are obtained through iteration. The end time of this stage can be divided into two situations:

- The end time *t*_2_ for emergency braking condition and ascend car overspeed is equal to the brake response time *t*_*br*_.
- The end time *t*_2_ under the condition of unintended car movement can be divided into two situations. If S1 stage ends:
  1. If the detection subsystem has not detected any unintended car movement, the end time *t*_2_ is the time *t*_*i*−1_ before the displacement of the car exceeds the detection distance *s*_*dd*_ in Eq. (21), plus the response time of the detection subsystem *tds*, the response time of the brakes *tbr*, and the end time of the S1 stage *t*1.
  2. The detection subsystem has detected the unintended car movement can. According to the calculation method in Eq. (16), the time required for the S1 stage car detection distance *s*_*dd*_ is calculated, plus the response time of the detection subsystem *t*_*ds*_ and the response time of the braking subsystem *t*_*br*_.

The specific equation is as follows:

22 $$\mathop t\nolimits_{2} = \left\{ {\begin{array}{*{20}l} {\text{EBC and ACO}} \hfill & {\mathop t\nolimits_{br} } \hfill \\ {{\text{UCM}}} \hfill & {\left\{ {\begin{array}{*{20}l} {\mathop t\nolimits_{i - 1} + \mathop t\nolimits_{ds} + \mathop t\nolimits_{br} + \mathop t\nolimits_{1} } \hfill & {\left| {\mathop x\nolimits_{{e_{{}} i - 1}} } \right| \le \mathop s\nolimits_{dd} {\text{ and }}\left| {\mathop x\nolimits_{{e_{{}} i}} } \right| > \mathop s\nolimits_{dd} } \hfill & {{\text{if }}\mathop {\left| {\mathop x\nolimits_{e1} } \right| \le s}\nolimits_{dd} \, } \hfill \\ {\mathop t\nolimits_{ds} + \mathop t\nolimits_{br} + \sqrt {\left| {\frac{{2\mathop s\nolimits_{dd} \left( {\mathop A\nolimits_{car} + \mathop A\nolimits_{cwt} } \right)}}{{\mathop B\nolimits_{cwt} - \mathop B\nolimits_{car} }}} \right|} } \hfill & {} \hfill & {{\text{if }}\mathop {\left| {\mathop x\nolimits_{e1} } \right| > s}\nolimits_{dd} } \hfill \\ \end{array} } \right.} \hfill \\ \end{array} } \right.$$

#### Brakes stopping traction sheave stage (S3 stage)

At this stage, the brakes of the traction machine begins to stop the traction sheave. Due to the fact that the brakes requires a relatively small amount of time to load to the maximum braking torque, the nonlinear least squares method is used to fit the traction machine braking torque test results of the prototype in the following text. A polynomial without a constant term about time *t* is constructed, and the torque obtained from the test results is represented by *b*. The coefficients of this polynomial can be calculated according to the following equation:

23 $$\mathop {\hat{c}}\nolimits_{{}} = \left( {A^{T} A} \right)^{ - 1} A^{T} b$$

**Fig. 8:**
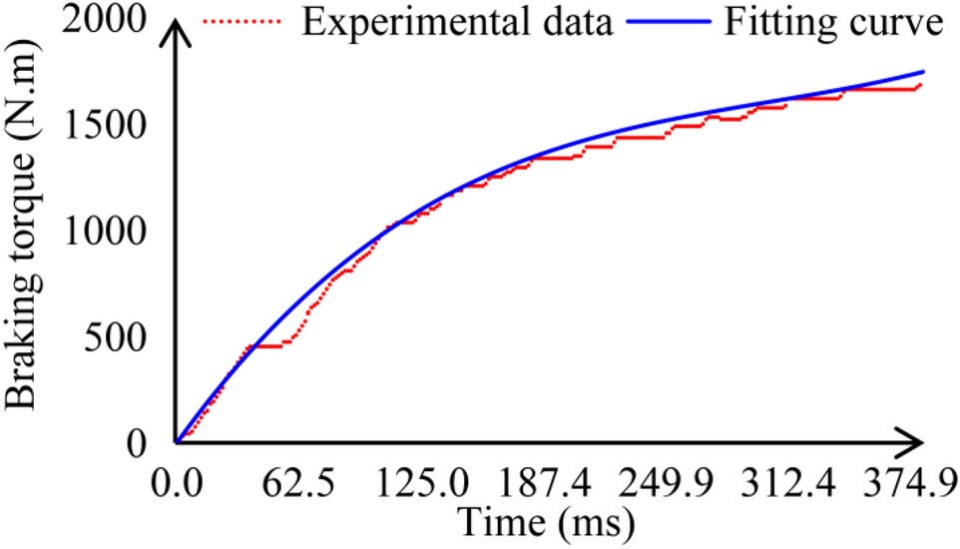
Braking torque test results and fitting diagram.

After comparative analysis, it was found that a third-order polynomial can fit the test results well, as shown in Fig. 8. Therefore, before the time required for the braking torque to be loaded to the maximum torque *t*_*tb*-max_, it can be represented by a third-order polynomial, followed by the maximum torque *T*_*b*-max_, as follows:

24 $$\mathop T\nolimits_{b} = \left\{ {\begin{array}{*{20}l} {\mathop {\hat{c}}\nolimits_{1} t + \mathop {\hat{c}}\nolimits_{2} t^{2} + \mathop {\hat{c}}\nolimits_{3} t^{3} } \hfill & {{\text{if }}t \le \mathop t\nolimits_{tb - \max } } \hfill \\ {\mathop T\nolimits_{b - \max } } \hfill & {{\text{if }}t > \mathop t\nolimits_{tb - \max } } \hfill \\ \end{array} } \right.$$

At this stage, the traction sheave begins to rapidly decelerate, and the suspension ropes also slows down. Generally, the reduced deceleration of the traction sheave will be greater than the deceleration provided by the traction force, so the suspension ropes will start to slip on the traction sheave.

The braking torque of the brakes is no longer 0. but needs to be calculated according to Eq. (24). By substituting the Eqs. (17) and (24) into Eq. (10), the differential equations for the reduced acceleration *a*_*m*_, reduced velocity *v*_*m*_ of traction shave and time* t* are obtained as follows:

25 $$ \mathop a\nolimits_{m} = \frac{{d\mathop v\nolimits_{m} }}{dt} = \frac{{\mathop B\nolimits_{cwt} - \mathop B\nolimits_{car} - \frac{{\mathop k\nolimits_{s} }}{{\mathop R\nolimits_{t} }}\left( \begin{gathered} \frac{3}{2}\mathop P\nolimits_{m} \frac{{\frac{{\left| {\mathop {rv}\nolimits_{m}\mathop P\nolimits_{m} } \right|}}{{\mathop R\nolimits_{t} }}\mathop \phi \nolimits_{f}^{2} \mathop R\nolimits_{s}^{3} + \left( {\frac{{\left| {\mathop {rv}\nolimits_{m}\mathop P\nolimits_{m} } \right|}}{{\mathop R\nolimits_{t} }}} \right)^{3} \mathop \phi \nolimits_{f}^{2} \mathop R\nolimits_{s} \mathop L\nolimits_{q}^{2} }}{{\left[ {\mathop R\nolimits_{s}^{2} + \left( {\frac{{\mathop {rv}\nolimits_{m}\mathop P\nolimits_{m} }}{{\mathop R\nolimits_{t} }}} \right)^{2} \mathop L\nolimits_{d} \mathop L\nolimits_{q} } \right]^{2} }} \hfill \\ + \left\{ {\begin{array}{*{20}l} {\mathop {\hat{c}}\nolimits_{1} t + \mathop {\hat{c}}\nolimits_{2} t^{2} + \mathop {\hat{c}}\nolimits_{3} t^{3} } \hfill & {{\text{if }}t \le \mathop t\nolimits_{tb - \max } } \hfill \\ {\mathop T\nolimits_{b - \max } } \hfill & {{\text{if }}t > \mathop t\nolimits_{tb - \max } } \hfill \\ \end{array} } \right. \hfill \\ \end{gathered} \right)}}{{\mathop A\nolimits_{car} + \mathop A\nolimits_{cwt} }} $$

The differential equation of the car acceleration *a*_*e*_ is equivalent to Eq. (19). Since the operating characteristics of the car at this stage are consistent with the previous section, the only difference is that the reduced acceleration of the traction sheave is not only related to *vm*, but also to the two independent variables *vm* and *t*. Therefore, it is only necessary to change *am*(*vm*) in Eq. (21) to *a*_*m*_(*t*, *v*_*m*_) to obtain the acceleration *a*_*e*3_, velocity *v*_*e*3_, and displacement *x*_*e*3_ of the car after the end of this stage according to the equations and methods in the previous section. After this stage is completed, the traction sheave stops rotating, so the reduced speed of the traction sheave is 0. The end time *t*_3_ can be calculated according to the following equation:

26 $$\mathop t\nolimits_{3} = \mathop t\nolimits_{2} + \mathop t\nolimits_{i - 1} \mathop {{\text{ When }}\mathop k\nolimits_{s} v}\nolimits_{{m_{{}} i - 1}} \ge 0{\text{ and }}\mathop {\mathop k\nolimits_{s} v}\nolimits_{{m_{{}} i}} < 0$$

#### Suspension ropes further sliding stage

After the traction sheave stops rotating, there are two operating states of the suspension ropes based on the car velocity *v*_*e*3_ at the end of stage S3:

- If *v*_*e*3_ = 0, it indicates that the suspension ropes stops together with the traction sheave.
- If *v*_*e*3_ ≠ 0, the suspension ropes will slip further on the traction sheave.

According to Eq. (14), the dynamic equation after the traction sheave stops rotating is obtained as follows:

27 $$\mathop a\nolimits_{e} = \frac{{d^{2} x}}{{dt^{2} }} = \mathop a\nolimits_{t} = \mathop k\nolimits_{s} \left( {\frac{{\left( {1 + \mathop k\nolimits_{s} } \right)\mathop B\nolimits_{car} }}{{2\mathop A\nolimits_{car} }} + \frac{{\mathop {\left( {1 - \mathop k\nolimits_{s} } \right)B}\nolimits_{cwt} }}{{\mathop {2A}\nolimits_{cwt} }}} \right)\left( {\frac{{\left( {\frac{{\mathop A\nolimits_{cwt} }}{{\mathop A\nolimits_{car} }}} \right)^{{\mathop k\nolimits_{s} }} + \left( {\frac{{\mathop B\nolimits_{cwt} }}{{\mathop B\nolimits_{car} }}} \right)^{{\mathop k\nolimits_{s} }} }}{{e^{{\frac{\lambda }{{10 + \left| {r\frac{dx}{{dt}}} \right|}}}} + \left( {\frac{{\mathop A\nolimits_{cwt} }}{{\mathop A\nolimits_{car} }}} \right)^{{\mathop k\nolimits_{s} }} }} - 1} \right)$$

Substitute the above equation into Eq. (20) and iteratively obtain the acceleration, velocity, and displacement of the car at different times during this stage. If the situation of *k*_*s*_*v*_*e i*+1_ > *k*_*s*_*v*_*e i*_ occurs during the iteration process, it indicates that the traction force is severely insufficient, causing the car to be unable to decelerate and requiring readjustment of elevator related parameters. Under normal circumstances, after this stage is completed, the car stops running, so the car velocity is 0. The end time *t*_4_ can be calculated according to the following equation:

28 $$\mathop t\nolimits_{4} = \mathop t\nolimits_{3} + \mathop t\nolimits_{i - 1} \mathop {{\text{ When }}\mathop k\nolimits_{s} v}\nolimits_{{e_{{}} i - 1}} \ge 0{\text{ and }}\mathop {\mathop k\nolimits_{s} v}\nolimits_{{e_{{}} i}} < 0$$

## Experimental verification

### Experimental procedure

This article uses emergency braking condition tests to verify the effectiveness of this method. The reason for not choosing other working conditions are:

- Due to the unintended car movement, the brakes cannot work before receiving the action command. After receiving the action command, the traction sheave must be stopped immediately. This process takes too short a time, making it difficult to simulate the actual situation and prone to accidents. Therefore, there are currently two main tests for testing the unintended car movement:
  1. Using UMCP testing mode, the control system sets a certain acceleration, and when the car reaches the detection point of the detection switch, it gives the brakes a command to stop the traction sheave to achieve the car’s stopping motion.
  2. After running at the maintenance speed for a certain distance, the car stops moving by cutting off the safety circuit to activate the brakes.
- Ascend car overspeed: When the car is running up, it reaches the electrical action speed of the speed governor, which cuts off the safety circuit and activates the brakes to stop the car from moving. Moreover, the ascend car overspeed condition is only applicable when the car is going up.

From the above, it can be seen that the testing principles for the other two operating conditions are basically the same as those for emergency testing, except for the initial speed. Because the dynamic characteristics of these three operating conditions are consistent in Stage 2–Stage 4, with only the initial speed being different, the effectiveness of this method can be fully verified through emergency braking condition tests. The main parameters of the prototype used in this experiment are shown in Table 1:

**Table 1:** Key parameters of the tested elevator.

Order number	Symbol	Value	Unit	Order number	Symbol	Value	Unit
1	*g*_*n*_	9.8	m/s^2^	10	*n*_*cr*_	2	
2	*Q*	2000	kg	11	*q*_*t*_	0.5	kg
3	*P*	1600	kg	12	*n*_*t*_	1	
4	*V*	1	m/s	13	*T*_*m*_	1682	N m
5	*r*	4		14	*t*_*br*_	0.25	S
6	*H*	51	m	15	*R*_*t*_	0.225	m
7	*q*_*sr*_	0.34	kg/m	16	*α*	161.368	°
8	*n*_*sr*_	6		17	*β*	105	°
9	*q*_*cr*_	3.82	kg	18	*γ*	25	°

For experimental verification, there are currently two main testing methods, one is HIL testing, and the other is to build a whole elevator for testing. Both testing methods have their own advantages and disadvantages^45,46^, since there are already elevators available for testing, the second method is adopted. For data collection, some scholars use graphical methods to collect the relative slip between the suspension ropes and the traction sheave^37,47^. However, to verify the calculation method in this paper, only the time-domain graphs of the acceleration, velocity, and displacement of the car need to be obtained. Therefore, the EVA-625, a specialized quality tester for elevators that can collect and analyze data on indicators such as acceleration, velocity, displacement, peak to peak vibration, and noise during elevator operation, was selected with a data collection time interval of 0.004S.

### Results

**Fig. 9:**
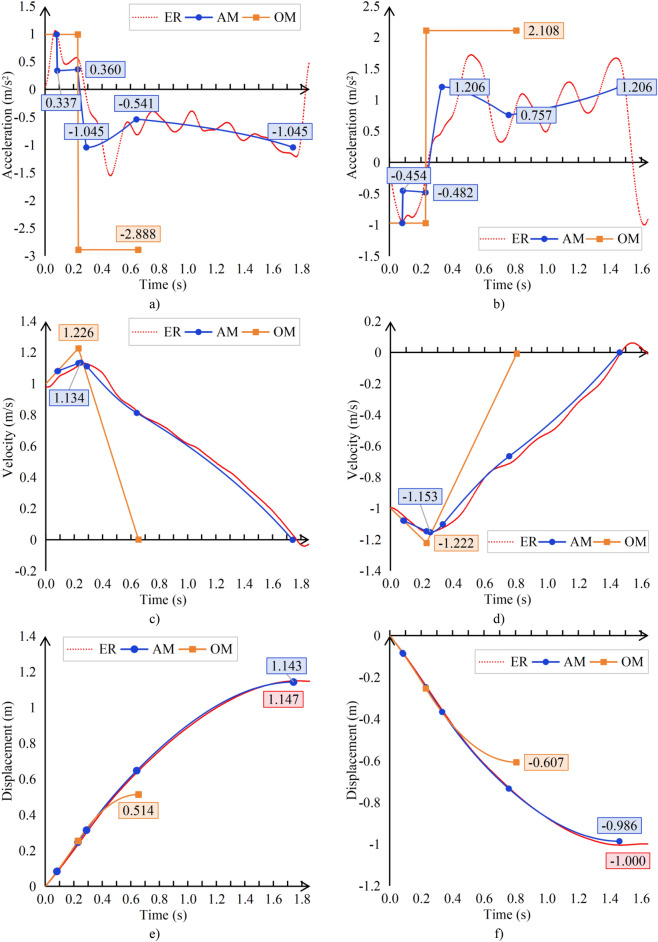
Comparison Chart (**a**) Upward acceleration 1 (**b**) Downward acceleration (**c**) Upward velocity (**d**) Downward velocity (**e**) Upward displacement (**e**) Downward displacement.

Based on the method described in this article (AM), the other method mentioned(OM) in the Sect.  1, and experimental data (ER), the acceleration, velocity and displacement results of the car are plotted on a graph as follows:

It can be clearly seen from Fig. 9 that the car stopping process can be divided into four stages:

- Free acceleration stage (S1 stage): The car begins to accelerate.
- Sealing torque intervention stage (S2 stage): After the intervention of the sealing torque, the acceleration is significantly reduced, but due to the small sealing torque provided by the sealing device in the low-speed and high-speed sections of the car, the car still accelerates slowly.
- Brakes stopping traction shear stage (S3 stage): After the brakes contacts the traction sheave, the acceleration of the car begins to be opposite to the running direction, and at the same time, the car begins to decelerate. However, due to the large deceleration of the traction sheave, the traction force of this sample elevator is no longer sufficient to support the synchronous deceleration of the suspension ropes with the traction sheave, and it begins to slide on the traction sheave.
- Suspension ropes further sliding stage (S4 stage): The traction sheave has stopped rotating, but the suspension ropes will slip on the traction sheave, but the slip speed is getting slower and slower, causing the speed difference between the suspension ropes and the traction sheave to decrease accordingly, resulting in an increase in the acceleration value.

From the displacement data of (e) and (f) in Fig. 9, the method proposed in this article is relatively close to the experimental data, while other method have significant errors compared to the experimental data. The calculation method in this article is 0.35% and 1.40% of the experimental data, which is close to the actual situation. The errors of other method are 55.19% and 39.3%, which are not in line with the actual situation.

## Further analysis

### Analysis of unintended car movement

#### Calculation results

Due to the release of the TSG T7007 code in 2022, all elevators applicable thereafter will be equipped with sealing devices. As shown in Fig. 8, the sealing torque increases rapidly at low speeds. Therefore, if there is a sealing device for protection, the unintended car movement will not be too large. However, as an independent protective device, the brakes must consider that the braking distance can meet the requirements in the event of a sealing device failure. Before the release of TSG T7007 standard, the mainstream machine room elevators in the market generally did not have sealing devices, so it is necessary to calculate the unintended car movement distance when the sealing torque is 0.

**Fig. 10:**
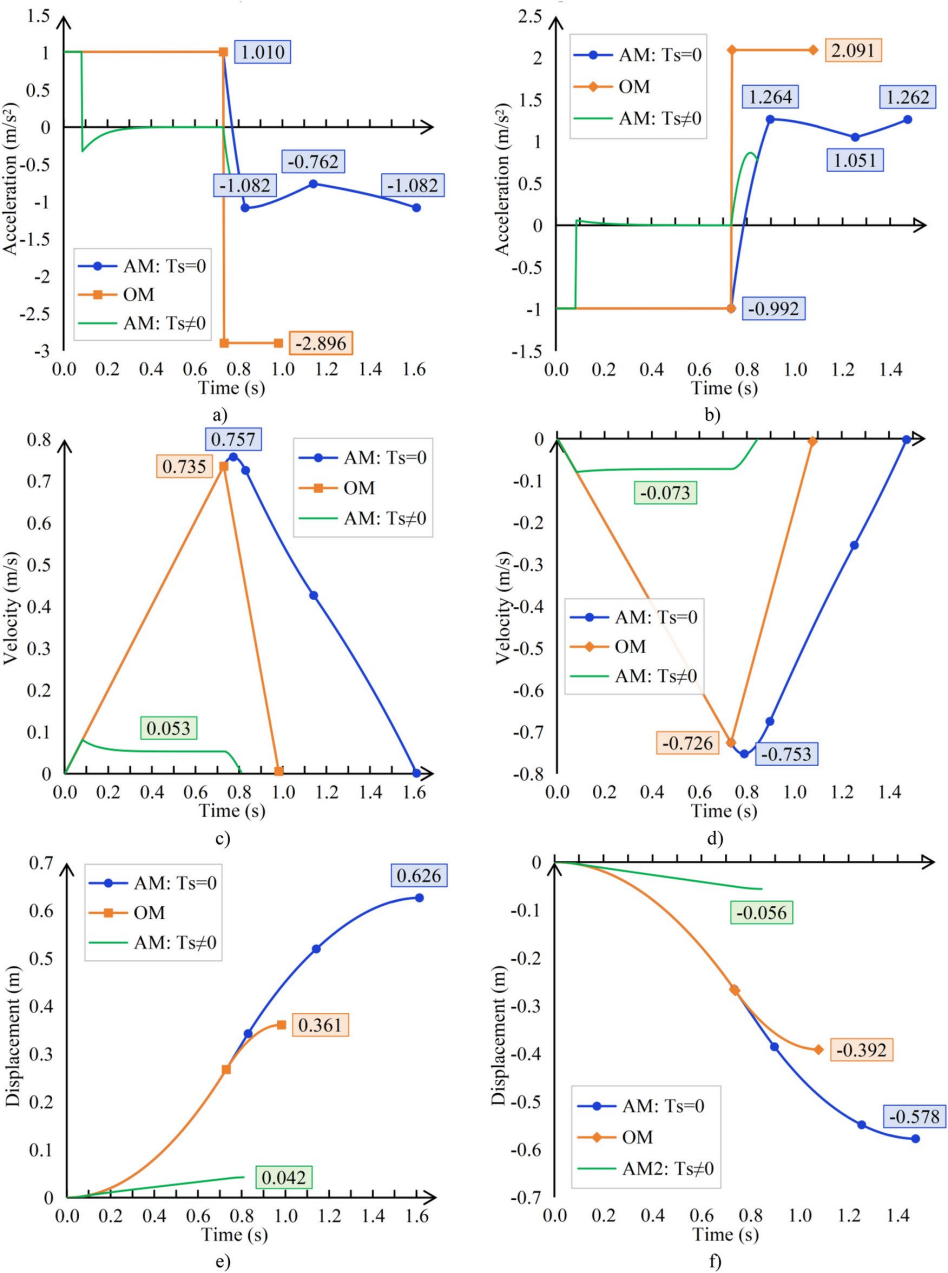
Comparison Chart (**a**) Upward acceleration 1 (**b**) Downward acceleration (**c**) Upward velocity (**d**) Downward velocity (**e**) Upward displacement (**e**) Downward displacement.

Figure 10 shows the braking acceleration, velocity, and displacement of the car when it is unloaded and moves upward on the bottom floor or when it is fully loaded and moves downward on the top floor.

It can be clearly seen from Fig. 10 that:

- If the further sliding of the suspension ropes on the traction sheave is not considered, the actual sliding distance will be far greater than the calculated distance, causing serious safety hazards.
- When the sealing torque *T*_*s*_ ≠ 0, the displacement of the car is very small.

#### Improvement of experimental methods

**Fig. 11:**
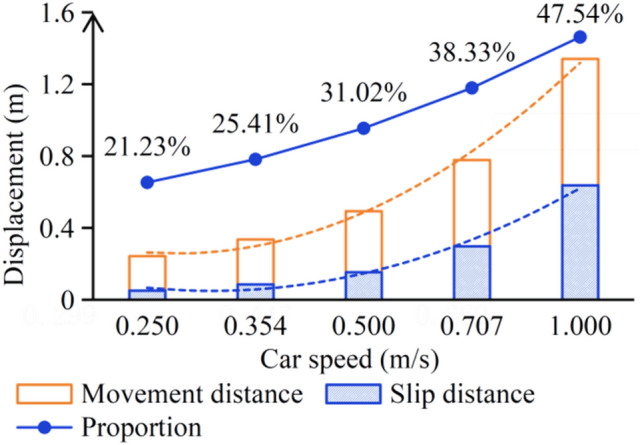
Calculation results of the car stopping distance.

According to the calculation equations, set 5 equally proportional speeds between 0.25 and 1.0 m/s, and calculate the car movement distance and sliding distance without the sealing device. The settlement result is shown in Fig. 11.

It can be clearly seen from Fig. 11 that as the speed increases, the stopping distance of the car will gradually increase non-linearly, and at the same time, the proportion of sliding distance of the suspension ropes will also increase. Therefore, it is not possible to calculate the car stopping distance at high speeds based on the speed ratio of the car stopping distance at low speeds. It is recommended to use the previous calculation method to calculate the velocity and displacement of the car when the brakes receives the command, and then conduct an emergency braking condition test at this velocity. Based on the test results, verify whether the free acceleration is correct and obtain the actual stopping distance, adding the previously calculated displacement, the actual stopping distance under unintended car movement can be roughly obtained.

### The influence of parameters on the results

#### Brake response time

**Fig. 12:**
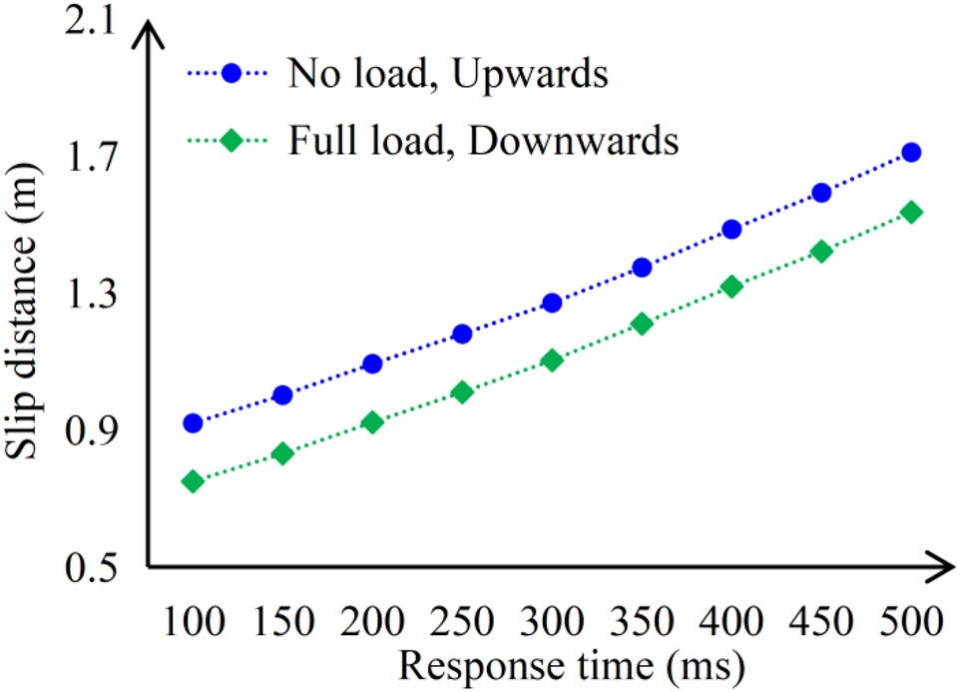
Diagram of the influence of brake response time on the distance of the car movement.

Research has found that the response time of the brakes has a significant impact on elevator deceleration and movement distance. Clause 4.3.2.6 of GB/T 24478-2003 only requires that the brake response time of the brakes should not exceed 0.5 s, specific requirements need to be discussed between elevator manufacturers and traction machine manufacturers ^48^. The actual response time of the traction machine brakes varies among different manufacturers^49^, the author investigated the response time data on the special equipment type test report of more than 10 traction machine brakes, which were distributed between 130 and 250 ms. According to the equation in the previous text, adjust the response time to obtain the elevator deceleration and movement distance at different response times, as shown in the following Fig. 12.

From Fig. 12, it can be seen that if the response time increases, the deceleration of the car becomes smaller and the deceleration distance becomes larger, until the car cannot decelerate and serious safety accidents such as roof collision occur. Therefore, it is recommended that elevator manufacturers negotiate with traction machine manufacturers to minimize brake response time based on actual conditions.

#### Sealing torque

From Fig. 7, it can be seen that the car speed starts from 0, and as the speed increases, the sealing torque rapidly increases, after reaching its maximum value, as the car speed increases, the sealing torque slowly decreases, indicating that the sealing torque has a relatively large influence on unintended car movement conditions and a relatively small impact on emergency braking and ascend car overspeed conditions. From Fig. 10, it can be seen that the effectiveness of sealing device has a significant influence on the stopping distance in the event of unintended car movement. If the sealing torque *T*_*s*_ ≠ 0, the force on the traction sheave reaches equilibrium in a very short time. At this time, the traction sheave drives the car to move up or down, so the speed of the car quickly enters a constant speed state.

**Fig. 13:**
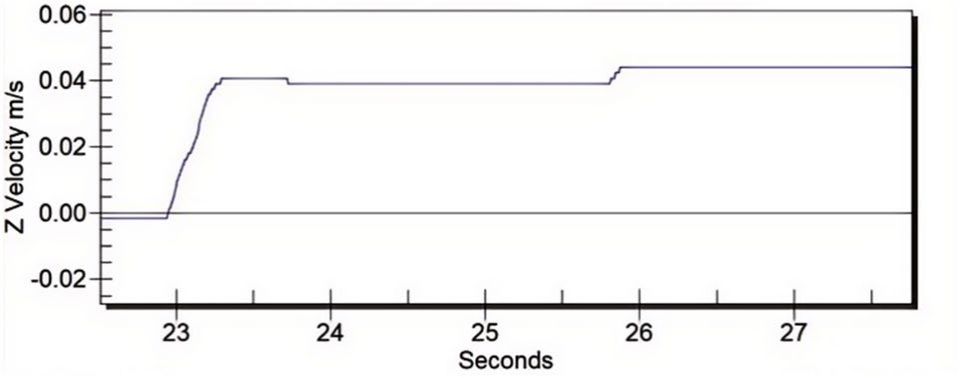
Car speed variation diagram under sliding test.

As mentioned in Sect. 4.1, it is not possible to conduct tests on unintended car movement. However, if only the speed change under the presence of the sealing torque is verified, the influence of sealing torque on the elevator car speed can be verified by conducting a car sliding test (braking torque *T*_*b*_ = 0) and observing the speed change of the car. Figure 13 is a screenshot of the PMT instrument’s monitoring record of the car speed. From Fig. 13, it can be seen that after a brief acceleration stage, the car speed enters a stable stage when it reaches approximately 0.05 m/s. Therefore, conducting unintended car movement test of the car starting from a stationary state without removing the sealing device has significant misleading results and cannot accurately reflect the actual situation.

#### The travelling height and car position

**Fig. 14:**
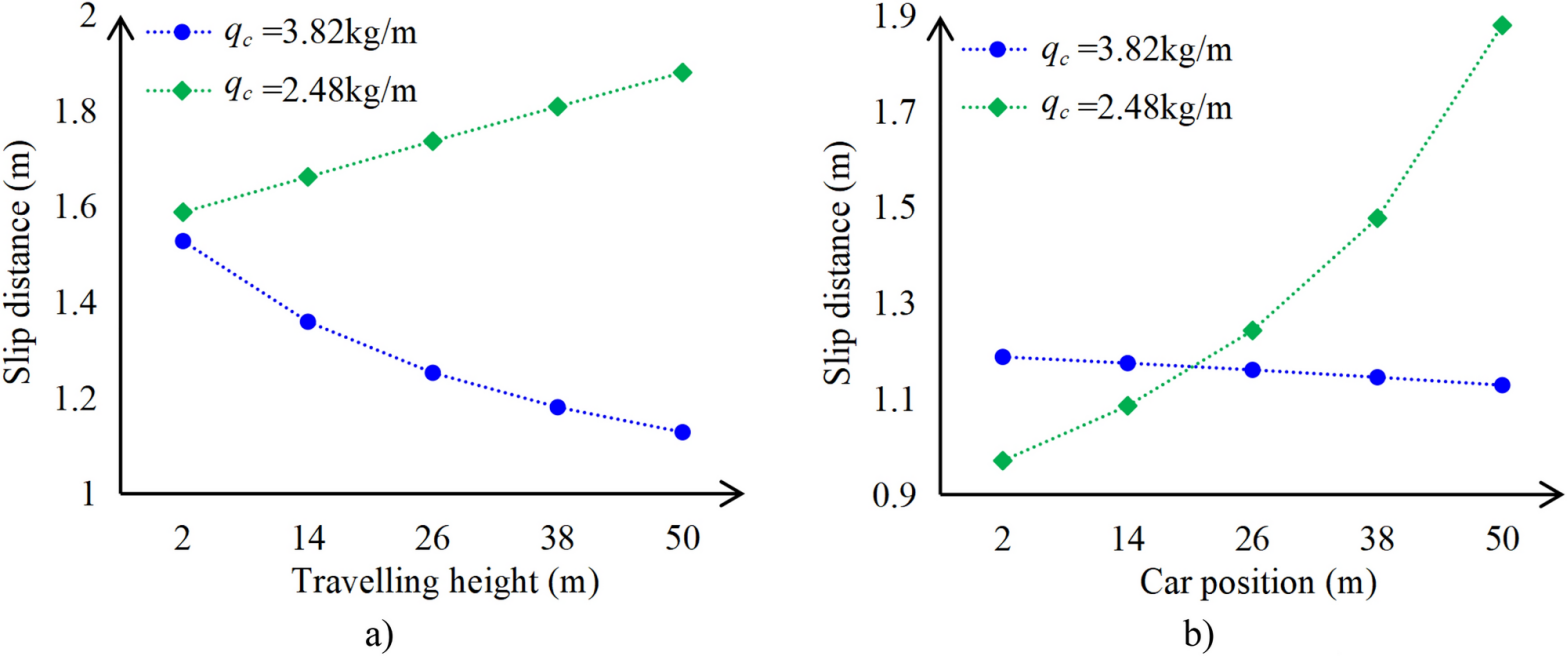
Comparison diagram of the car stopping distance (**a**) Change in travelling height (**b**) Change in car position.

The travelling height *H* of each elevator order is generally different, and the elevator will inevitably be in different positions during its up and down operation. Therefore, analyze the influence of changes in travelling height and car position on the stopping distance of the car. Figure 14 shows the braking distance of the car under emergency braking conditions when it is moving up without load, at different travelling heights or positions.

From Fig. 14, it can be seen that as long as the specifications of the compensation chains change, the stopping distance of the car at the maximum travelling height or the topmost position may not be the maximum. Therefore, when designing elevators, it is necessary to consider the braking distance of the car at different positions within the travelling height range.

#### Other parameters

**Fig. 15:**
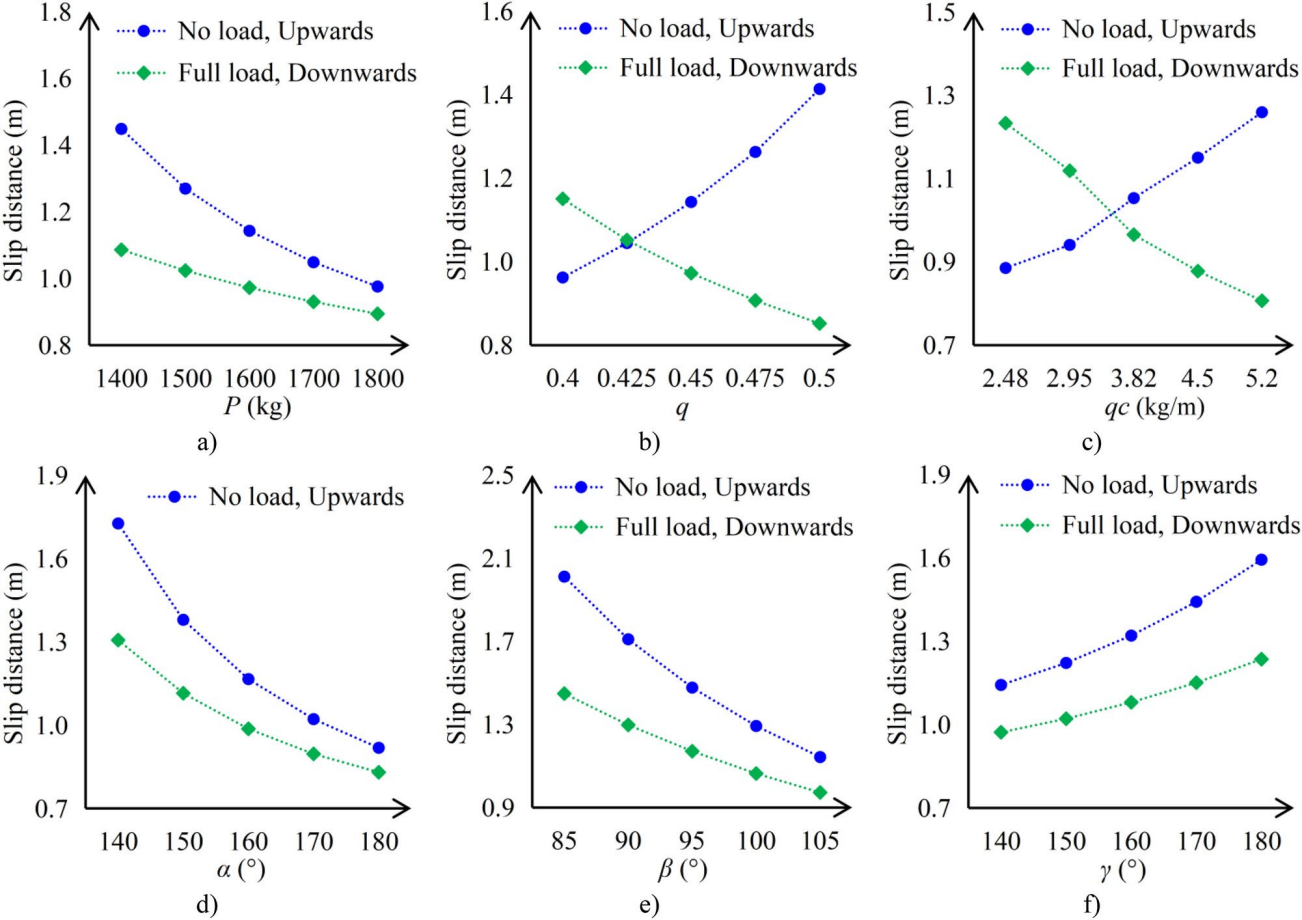
The influence of parameters on the results (**a**) the car mass. (b) The elevator speed. (**c**) The balance factor. (**d**) The angle of wrap of the ropes on the traction sheave. (**e**) The value of the undercut angle. (**f**) The value of the groove angle.

After the completion of elevator development, in general, the car mass *P*, Compensation chain weight per meter *q*_*c*_, the value of the undercut angle *β*, the value of the groove angle *γ*, and the angle of wrap of the ropes on the traction sheave *α*, will be changed according to specific order requirements. Each elevator manufacturer generally only specifies a range for the balance factor *q*, which is determined by the on-site installation situation. Therefore, the influence of these parameters on the results is analyzed, as shown in Fig. 15.

From Fig. 15, it can be seen that the sliding distance of the elevator car is:

- Directly proportional to the value of the groove angle.
- Inversely proportional to the car mass, the value of the undercut angle, and the angle of wrap of the ropes on the traction sheave.
- Reducing the weight of the compensation chain will increase the stopping distance of the car when it is unloaded and going up, but will decrease the stopping distance of the car when it is fully loaded and going down, and vice versa.
- When the car is running upwards with no load, the sliding distance of the car is proportional to the balance factor; when the car is running downwards with full load, the sliding distance of the car is inversely proportional to the balance factor.

When designing elevators, elevator manufacturers can adjust the above parameters according to the actual situation to change the sliding distance of the car, ultimately improving the safety performance of the elevator.

## Conclusion

- The analysis of elevator operating characteristics shows that before the traction machine brakes brake the traction sheave, the elevator will continue to accelerate until the sealing torque intervenes, and the acceleration of the car will significantly decrease. However, it generally still accelerates slowly and will not directly enter the deceleration stage. After the car decelerates, the suspension ropes and traction sheave begin to decelerate synchronously. Due to the fact that the reduced deceleration of the traction sheave will quickly exceed that of the car, the traction force will begin to be insufficient to support the synchronous deceleration of the car and traction sheave, and the suspension ropes will start to slip on the traction sheave. After the traction sheave stops, the suspension ropes will continue to slide on the traction sheave until the car stops.
- This article establishes new differential equations for acceleration, velocity, and displacement of the car, and proposes an approximate method for solving the time-varying equations of acceleration, velocity, and displacement of the elevator car. Through experimental verification, the curve calculated by the method in this article is basically consistent with the actual situation. From the displacement situation, the theoretical and practical error has been reduced from about 39% to about 1.5%.
- Analysis and experiments have shown that the sealing torque has a significant impact on the low-speed operation of the car, and the slip dynamics of the suspension ropes on the traction sheave indicate that the faster the car speed, the greater the slip distance of the suspension ropes. Therefore, the current experiment on unintended car movement at low speeds cannot accurately reflect the actual stopping distance. By calculating the car velocity and displacement when the brake receives the command, and then conducting emergency braking condition tests at this velocity, the actual stopping distance under unintended car movement can be indirectly obtained.

## Data Availability

The datasets used and analyzed during the current study are available from the first author on reasonable request.

## List of symbols

*g*_*n*_: The standard acceleration of free fall, m/s^2^
*r*: The reeving factor
*P*: The masses of the empty car, kg
*Q*: The rated load, kg
*q*: The balance factor
*T*_*car*_: Tension of the suspension ropes on the car side at the traction sheave, N
*T*_*cwt*_: Tension of the suspension ropes on the counterweight side at the traction sheave, N
*f*: Equivalent friction coefficient between suspension ropes and traction sheave
*a*_*m*_: Reduced acceleration of the traction sheave: the acceleration of the traction sheave divided by the reeving factor *r*, m/s^2^
*v*_*m*_: Reduced velocity of the traction sheave: the linear velocity of the traction sheave divided by the reeving factor *r*, m/s
*a*_*t*_: Maximum car acceleration provided by traction force, m/s^2^
*a*_*e*_: Acceleration of the elevator car, m/s^2^
*v*_*e*_: Velocity of the elevator car, m/s
*M*_*cwt*_: The mass of counterweight including mass of pulleys, kg
*M*_*load*_: Car loading weight, kg
*H*: The travel height, m
*H*_*car*_: The position of the elevator car in the well, m
*M*_*SRcar*_: The mass of the suspension ropes on the car side, kg
*M*_*SRcwt*_: The mass of the suspension ropes on the counterweight side, kg
*L*_*SRcar*_: When the elevator car moves to the top floor, the length of the suspension ropes between the traction sheave and the elevator car, m
*L*_*SRcwt*_: When the car moves to the bottom floor, the length of the suspension ropes between the traction sheave and the counterweight, m
*n*_*s*_: Number of the suspension ropes
*q*_*s*_: The mass per meter of the suspension ropes, kg/m
*M*_*CRcar*_: The mass of the compensation chains on the car side, kg
*M*_*CRcwt*_: The mass of the compensation chains on the counterweight side, kg
*L*_*CRcar*_: When the car moves to the bottom floor, the length of the compensation chains on the car side, m
*L*_*CRcwt*_: When the elevator car moves to the top floor, the length of the compensation chains on the counterweight side, m
*n*_*c*_: The number of the compensation chains
*q*_*c*_: The mass per meter of the compensation chains, kg/m
*M*_*Trav*_: The mass of the traveling cables on the car side, kg
*L*_*Trav*_: When the elevator car moves to the top floor, the length of the traveling cable on the elevator car side, m
*n*_*t*_: The number of the traveling cables
*q*_*t*_: The mass per meter of the traveling cables, kg/m
*FR*_*car*_: The frictional force in the well (efficiency of bearings car side and friction on guide rails), N
*FR*_*cwt*_: The frictional force in the well (efficiency of bearings counterweight side and friction on guide), N
*J*_*DPcar*_: Inertia of the pulleys on the car side, kg·m^2^
*J*_*DPcwt*_: Inertia of the pulleys on the counterweight side, kg·m^2^
*J*_*Pcar*__-__*i*_: The inertia of a single pulley with the same rotational speed at the i-th position on the car side, kg·m^2^
*J*_*Pcwt*__-__*i*_: The inertia of a single pulley with the same rotational speed at the i-th position on the counterweight side, kg·m^2^
*i*_*Pcar*__-__*i*_: The number of pulleys with the same rotational speed at the i-th position on the car side
*i*_*Pcwt*__-__*i*_: The number of pulleys with the same rotational speed at the i-th position on the counterweight side
*α*: The angle of wrap of the ropes on the traction sheave, °
*β*: The value of the undercut angle, °
*γ*: The value of the groove angle, °
*T*_*b*_: Traction machine brakes torque, N m
*T*_*s*_: Traction machine sealing torque, N m
*t*_*br*_: Response time of the traction machine brakes, s
*t*_*st*_: Response time of the sealing devices, s
*R*_*s*_: Stator resistance, Ω
*P*_*m*_: Number of motor poles
$\varphi$_*f*_: Rotor magnetic flux, V s
*L*_*d*_: Cross axis inductance, H
*L*_*q*_: Direct axis inductance, H
*t*_*ds*_: Response time of the detection subsystem, sec
*E*_*r*_: Elastic modulus of the suspension ropes, N/m^2^
